# Database Analysis of Application Areas and Global Trends in Ketogenic Diets from 2019 to 2024

**DOI:** 10.3390/nu17091478

**Published:** 2025-04-27

**Authors:** Marc Assmann, Isabel Albrecht, Marius Frenser, Thorsten Marquardt, Tobias Fischer

**Affiliations:** 1 Center for Nutrition and Therapy (NuT), University of Applied Sciences Muenster, Corrensstraße 25, 48149 Muenster, Germany; 2 Department of Pediatrics, University Hospital Muenster, Albert-Schweitzer-Campus 1, 48149 Muenster, Germany

**Keywords:** ketogenic diet, ketogenic nutrition, research areas, nutrition trends, global trends, database analysis, literature analysis

## Abstract

**Background**: After being developed in the 1920s, the ketogenic diet fell into disuse, only to make a comeback at the end of the 20th century. In addition to its original use in the treatment of epilepsy, research on the ketogenic diet is now focusing on many other indications. **Methods**: Based on a systematic literature analysis according to the PRISMA guidelines, an overview of the current research on specific topics in the last five years (2019 to August 2024) was compiled. **Results**: A total of 290 trials were included. In total, 32 topics were analyzed, most of which were related to overweight and obesity, as well as exercise and epilepsy. The articles included 1981 authors from 47 countries, who published their results from intervention and observational studies in 153 journals. In total, 227 studies lasted less than six months, while 61 studies lasted more than six months. **Conclusions**: The results and the increasing amount of research underline the growing scientific attention and potential of the ketogenic diet to offer new therapeutic and individual preventive approaches. These trends indicate that the ketogenic diet remains an important international research topic.

## 1. Introduction

The ketogenic diet (KD) is a very high fat, very low carbohydrate diet that mimics the state of fasting by reducing plasma glucose and insulin levels while increasing lipid metabolism [1,2]. The induced ketogenesis leads to the formation of the ketone bodies β-hydroxybutyrate (ßHB), acetoacetate (AcAc), and acetone. The first two, ßHB and AcAc, serve as an alternative energy source to glucose in the fasting state, during vigorous exercise, or as part of a KD [3,4,5,6]. Acetone, which is formed through the spontaneous decarboxylation of AcAc, does not play a role in energy supply [7]. An increase in blood ketone bodies, mainly ßHB [8,9], is called ketosis (physiological: 0.5–8 mmol/L, diabetic ketoacidosis: 15–25 mmol/L [10,11]). The extent of ketosis varies depending on the duration of fasting, the type of KD, or other ketosis-promoting interventions such as exogenous ketones (e.g., ketone esters or salts) [12,13,14,15,16].

Indications for a KD primarily include pharmacoresistant epilepsy in children and adolescents, as well as certain congenital metabolic disorders, such as glucose transporter Type 1 deficiency and pyruvate dehydrogenase deficiency [17,18,19]. The KD was founded in 1921 by Dr Wilder, an American physician at Mayo Clinic in Rochester, Minnesota, in the context of seizure reduction in children with epilepsy. Wilder suspected that ketone anemia, the increased presence of ketone bodies in the blood, could be induced by mimicking a fasting state rather than by actual starvation, which often led to rapid therapy discontinuation. Four years later, Peterman, who also worked at Mayo Clinic, developed the classic KD for children and adolescents, which restricted carbohydrate intake to 10–15 g per day [1,20,21]. The classic KD is based on a ketogenic ratio, i.e., the ratio of fat to the sum of carbohydrates and proteins in grams, ranging from 3:1 to 4:1. Other well-known forms of ketogenic dietary therapies include the modified Atkins diet (MAD), the medium-chain triglyceride diet (MCTD), and low glycemic index therapy (LGIT). The classic KD is the most restrictive form, producing the highest rate of ketosis, but is the most difficult to implement in practice due to its strict limitations [22,23].

With the development of new antiepileptic drugs in the 1930s, the popularity of the KD rapidly declined, and fewer dietitians were trained in its implementation. It was not until the 1990s that interest was revived after Freeman and Kelly, a physician and a nutritionist at Johns Hopkins University in Baltimore, USA, successfully treated a two-year-old child with severe pharmacoresistant epilepsy using the KD. The boy’s father subsequently founded the Charlie Foundation, which continues to support affected families and medical professionals with information on the KD [20,24]. The growing interest and subsequent increase in research over the last few decades have identified new potential fields of application for the ketogenic diet. These include cancer, obesity, diabetes mellitus, neurodegenerative diseases, chronic kidney disease, competitive sports, and modulation of the microbiome [7,25,26,27].

A bibliometric analysis by Ye et al. [26] in the Web of Science database from 2001 to April 2022 analyzed the number of annual studies and global trends in the KD. It was found that until 2012, fewer than 100 studies were published per year, whereas from 2019 onward, there was a sustained increase of more than 200 studies per year. Over the past decade, the focus has broadened to include topics such as cancer and fatty liver, inflammatory, and mitochondrial diseases. This reflects an increased interest in research in this area. The aim of this database analysis is to provide a current overview of ketogenic dietary approaches, including both established applications and emerging trends, over the past five years. Additionally, it aims to highlight existing research priorities and to identify potential new areas of research.

## 2. Materials and Methods

### 2.1. Data Acquisition

The two online databases PubMed and ScienceDirect were used to obtain the data. For data acquisition, a search string was created for PubMed and ScienceDirect based on the characteristics of a KD, as well as synonyms for KD and MCT (see Table A1). The search was performed for PubMed on 16 August 2024 and for ScienceDirect on 19 August 2024. Studies published between January 2019 and August 2024 were included, and additional applied filters are listed in Appendix A. To ensure a systematic approach, the data collection process followed the PRISMA guidelines for systematic reviews [28]. Inclusion criteria included both the presence of a KD (carbohydrate restriction to a maximum of 60 g/day or the presence of measured ketosis > 0.5 mmol/L) and controlled and uncontrolled study designs on the KD. Reviews, meta-analyses, case studies, and all non-research articles such as editorials, letters, and commentaries were excluded.

After duplicate removal using the reference management software Citavi version 6.19.1, the hits were checked for accuracy using title and abstract, and then, the full text was analyzed. The included articles were then sorted by topic areas in an Excel spreadsheet (version 2441), and data from the abstract and full text were extracted. The following data were extracted from the publications: journal title, indication or application area of the KD, year of publication, study design, number of subjects, gender distribution of subjects, dropouts, study duration, names of authors, and country of first author at time of publication. The number of citations (as of 7 January 2025) and keywords used were extracted from PubMed. Data on the impact factor were obtained from the journals’ websites. To ensure data quality, data extraction was performed independently by two nutritionists.

### 2.2. Statistical Analysis and Visualization

For the statistical calculations, methods of descriptive statistics, such as mean, median, standard deviation, and range, were calculated using Microsoft Excel for Microsoft 365 MSO (version 2441). Microsoft Word and Excel for Microsoft 365 MSO (version 2441), the bibliometric software tool VOSviewer (version 1.6.20), and the web-based word cloud tool from WordArt.com (version 4.30.0) were used for graphical illustrations and tables.

## 3. Results

### 3.1. Search Results

**Figure 1:**
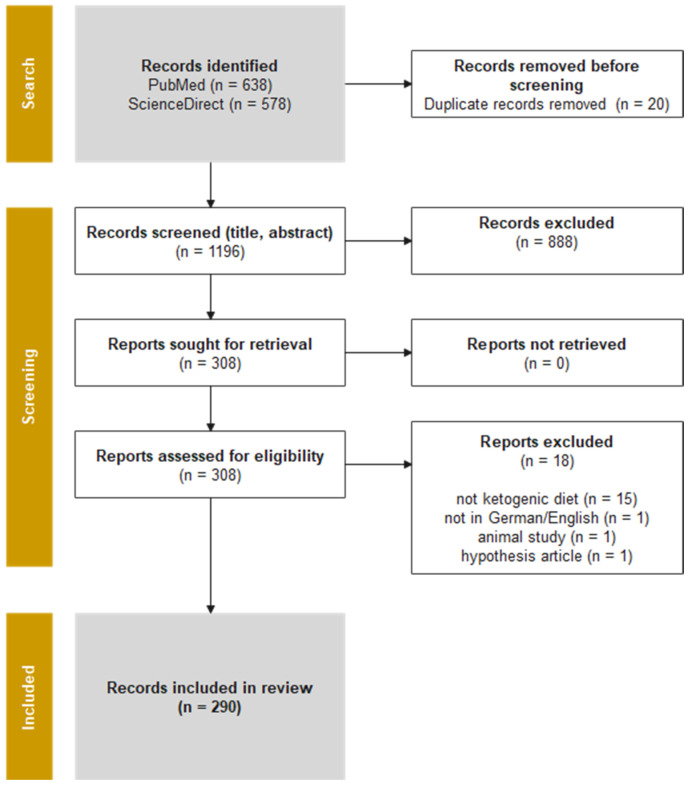
A flow chart of the results of the database search (PubMed, ScienceDirect) based on the PRISMA guideline for systematic reviews (modified from [28]).

Based on the search strings (see Appendix A), a total of 1216 records in the field of ketogenic nutrition were identified via PubMed and ScienceDirect. After merging these results, a total of 20 duplicates (1.64%) were removed. A further 888 articles (73.03%) were removed after title and abstract screening due to unsuitable study types. Another 18 articles (1.48%) were excluded because they did not meet the inclusion criteria. In total, 290 studies (23.85%) were included (see Figure 1). References to all 290 included studies are provided in Supplement S1.

### 3.2. The Research Areas of the KD

**Figure 2:**
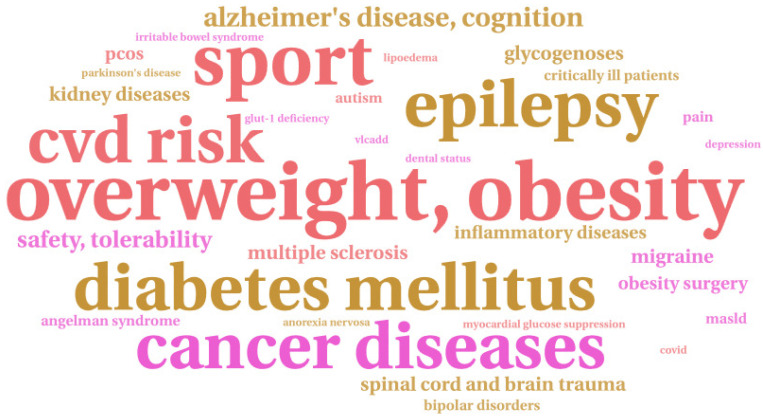
A word cloud of topic areas of extracted publications (PubMed, ScienceDirect) related to a KD in the period from 2019 to August 2024. Created with WordArt.com.

A total of 32 different topics were identified from the included studies (see Figure 2). The most common topic was overweight and obesity (n = 56, 19.31%), followed by epilepsy (n = 37, 12.76%), sports (n = 36, 12.41%), diabetes mellitus (n = 31, 10.69%), cancer (n = 28, 9.66%), cardiovascular disease (n = 21, 7.24%), Alzheimer’s disease and cognition (n = 15, 5.17%), the safety and tolerability of appropriate diets (n = 8, 2.76%), spinal cord and brain trauma (n = 5, 1.72%), and migraine (n = 5, 1.72%) (see Table 1). In total, 22 topics were included in fewer than five trials (each) (n = 57, 19.66%).

### 3.3. Study Designs

**Figure 3:**
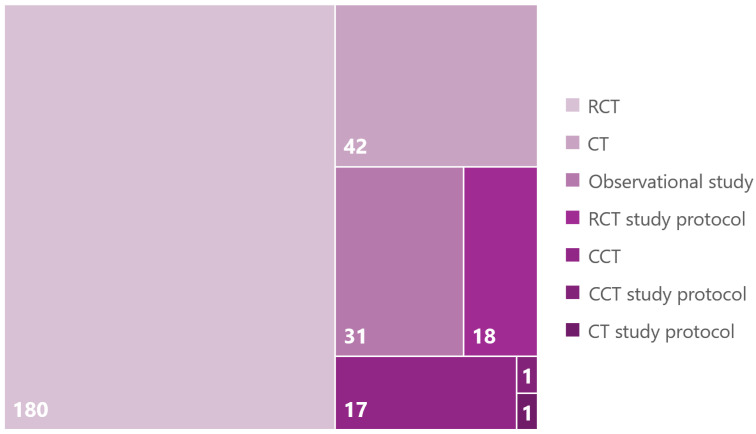
The number (N = 290) of study designs or study protocols of publications extracted from PubMed and ScienceDirect from 2019 to August 2024. Classification according to PubMed categorization.

The included studies were mainly completed randomized controlled trials (RCTs) (n = 180, 62.1%) or study protocols for RCTs (n = 18, 6.21%) (see Figure 3). Following by a large margin were clinical trials (CTs) (n = 42, 14.48%), study protocols of clinical trials (n = 1, 0.34%), observational studies (n = 31, 10.69%), study protocols of observational studies (n = 1, 0.34%), and controlled clinical trials (CCTs) (n = 17, 5.86%).

### 3.4. Annual Publications

**Figure 4:**
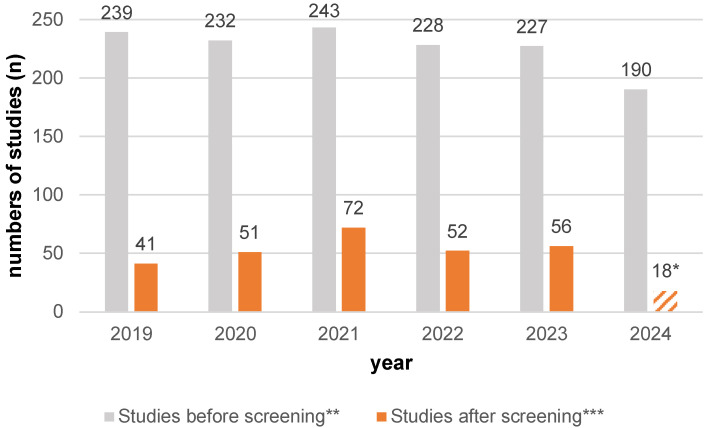
The number of studies (N = 290) per year in the analyzed period from 2019 to August 2024 (orange) and the total number of studies according to the search strategy (gray; see Table A1) in the online literature databases PubMed and ScienceDirect. * Only considered up to and including August 2024. ** The total number of studies according to the search strategy (see Table A1). *** The number of studies according to the search strategy (see Table A1) after PRISMA screening (see Figure 1).

The number of published articles included in this overview as part of the PRISMA analysis (N = 290, see Figure 1) varied over the five-year period analyzed (see Figure 4, orange column). Initially, the number of publications increased from 2019 (n = 41, 14.14%) through 2020 (n = 51, 17.59%) to a maximum in 2021 (n = 72, 24.83%). This was followed by a decline in the next two years, with approximately 50 publications per year (n = 52, 17.93%; n = 56, 19.31%). The year 2024 was only included until August and contained 18 publications (6.21%).

### 3.5. Duration of Studies

**Figure 5:**
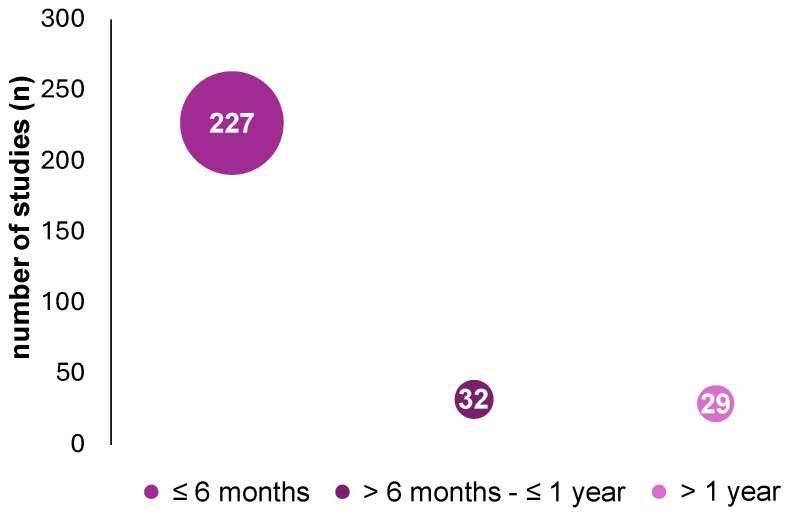
Distribution of KD studies from PubMed and ScienceDirect from 2019 to August 2024 (n = 288) by study duration ≤ 6 months (n = 227), >6 months to ≤1 year (n = 32), and >1 year (n = 29).

For the evaluation of study duration (see Figure 5), 288 of the 290 studies could be included, because the remaining studies either did not provide data (n = 1) or were planned to run until the end of the subjects’ lives, and, therefore, the final information was not available (n = 1). The duration of the included studies varied between a minimum of one day and a maximum of 18 years (217.79 ± 494.87 days; median: 90 days). Studies of ≤6 months accounted for the largest proportion (n = 227, 78.82%), followed by studies of >6 months to 1 year (n = 32, 11.11%). Only 10.1% of studies (n = 29) lasted longer than one year.

### 3.6. Number of Subjects and Gender Distribution

The number of included subjects could be extracted from all 290 studies (n = 151,856; mean: 523 ± 7389.68; median: 37) (see Table 2). The study by Titcomb et al. [29] was an outlier due to its prospective observational design, as 125,982 subjects were included in the data analysis. Excluding the number of subjects from Titcomb et al., the total number of subjects amounts to 25,874 (89.53 ± 380.71; median: 37).

Gender information was available for 223 trials (77%). A total of 133,504 women (598.67 ± 8415.36; median: 15) and 5958 men (26.72 ± 37; median: 14) participated in the studies. Excluding the study by Titcomb et al., which included only women, the number of female participants was 7522 (33.88 ± 56.13; median: 15). This results in a sex ratio of 22.41:1 (female/male) with and 1.26:1 (female/male) without the data from Titcomb et al.

The number of dropouts (total) was obtained from 174 publications. A total of 2728 subjects (23.54%; mean: 15.61 ± 34.84; median: 5) dropped out prematurely after inclusion in the studies. Data were also available on dropouts of the female sex in 77 studies, of which 15 included only women, and of the male sex in 71 studies, of which 9 included only men. Retention was similar for women (87.64% ± 16.02%; median: 93.65%) and men (87.28% ± 18%; median: 100%).

### 3.7. An Analysis of the Authors

**Figure 6:**
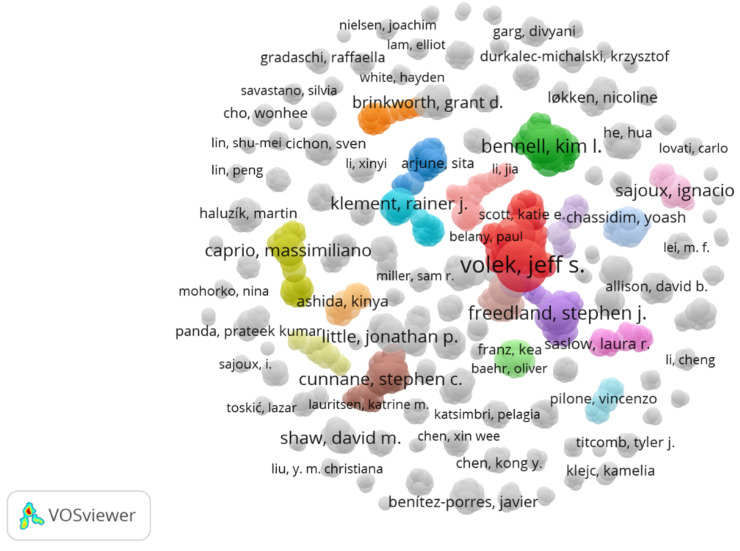
A bubble chart of author collaborations related to publications on the KD from 2019 to August 2024 in the online literature databases PubMed and ScienceDirect.

A total of 1981 different authors are named in the 290 included studies, regardless of the position of their citation. An overview of the most relevant authors and existing collaborations is shown in Figure 6. Most publications were authored by Jeff S. Volek (n = 14), followed by Alex Buga (n = 9), Christopher D. Crabtree (n = 7), Madison L. Kackley (n = 7), and Parker N. Hyde (n = 7). Table 3 lists all authors with ≥6 publications during the analysis period. There are 1615 authors mentioned with only one publication.

### 3.8. A Breakdown of the Authors by Country

**Figure 7:**
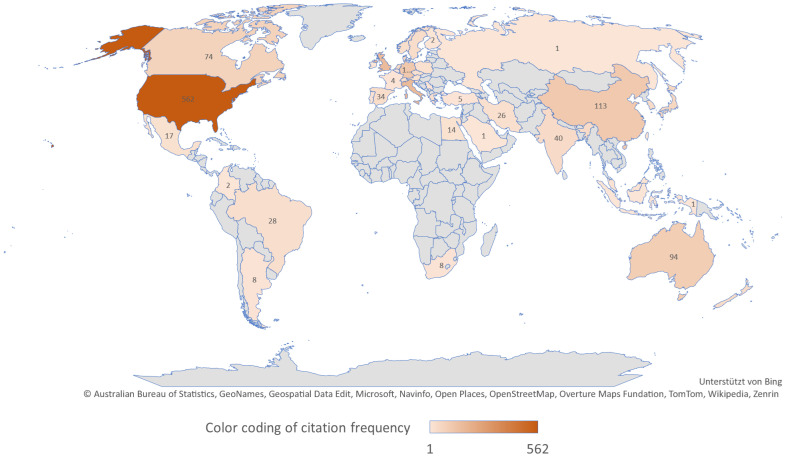
A map of the country distribution (n = 47) of authors (n = 1981) of publications on the KD with color coding of citation frequency (the darker the brown, the more frequently cited) in the period from 2019 to August 2024 in the online literature databases PubMed and ScienceDirect.

The 1981 authors lived or worked in 47 different countries at the time of publication (42.64 ± 86.73; median: 17). Based on the publications (N = 290), a total of 2004 countries, including 23 with information on two countries, could be assigned to the named authors. The most frequently mentioned country was the USA (n = 562, 28.37%), followed by Italy (n = 175, 8.83%) and the United Kingdom (n = 169, 8.53%). Seven countries, including Belgium, Indonesia, and Russia, were represented only once across all publications (see Figure 7). Table 4 shows the top 10 most represented countries.

### 3.9. Journals

The publications (N = 290) appeared in 153 different journals. Most studies were published in *Nutrients* (n = 47), followed by *Clinical Nutrition* (n = 10) and *BMC Trials* (n = 9). The 13 most frequently used journals and their impact factors from 2023 are shown in Table 5. The majority of journals (n = 113) published only one study on the KD.

### 3.10. Citations

According to PubMed, the publications (N = 290) were cited in a total of 4143 other articles (14.29 ± 21.92; median 6). The publications by Paoli et al. (n = 153, 3.69%), Lowe et al. (n = 121, 2.92%), Ota et al. (n = 110, 2.66%), Khodabakhshi et al. (n = 107, 2.58%), and Martins et al. (n = 104, 2.51%) were most frequently cited. A total of 176 articles (60.69%) received fewer than 10 citations, while 25 articles (8.62%) were not cited at all. Table 6 presents the five most frequently cited articles.

### 3.11. Keywords

**Figure 8:**
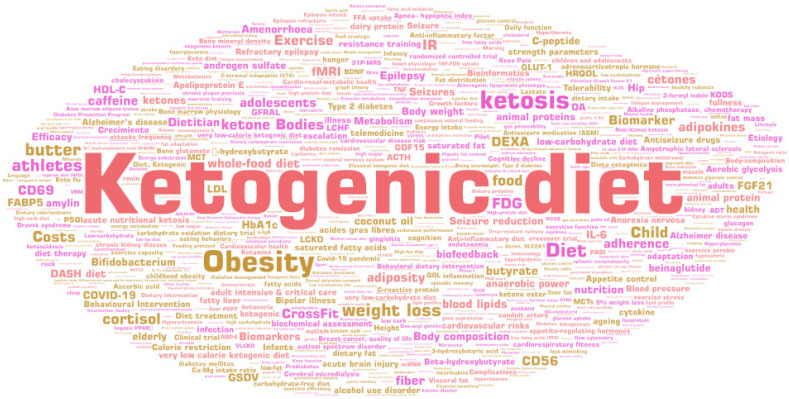
A word cloud for analyzed keywords (n = 760) in the included publications from 2019 to 2024 in the online literature databases PubMed and ScienceDirect. The size of the keyword shown correlates with the number of mentions (maximum n = 91). Created with WordArt.com.

Based on the abstract-based keyword analysis in PubMed, 243 publications (84%) were included. In 47 articles (16%), no keywords were given in PubMed. A total of 760 different keywords were used. The most common term was “ketogenic diet” (n = 91, 37.45%), followed by “obesity” (n = 32, 13.17%), “ketosis” (n = 27, 11.11%), “diet” (n = 21, 8.64%), and “weight loss” (n = 19, 7.82%) (see Figure 8). In total, 576 keywords were mentioned at least once. The most common keywords mentioned in ≥10 publications are shown in Table 7.

## 4. Discussion

The number and frequency of publications in the various research areas over the five years examined showed slight fluctuations with no discernible trend. However, certain topics showed anomalies in publication patterns. For example, the number of studies in the field of oncology increased steadily from two to twelve studies per year from 2019 to 2021. However, in 2022, only five studies were published, with no further new studies following this. A review of the KD in relation to cancer showed an increase in articles from 2012 (n = 20) to a maximum in 2020 (n = 103) and a slight decrease in 2021 (n = 96) [35]. Differences with the present study could be due to the inclusion of reviews, searches in other databases, and the inclusion of studies in which non-ketogenic interventions were performed. In contrast, the number of studies on cardiovascular risk increased continuously until 2023. Studies on bipolar disorder were first published in 2023 and 2024, and studies on depression and Parkinson’s disease in 2024. Bibliometric reviews identified the first studies on neurological diseases such as Parkinson’s disease and amyotrophic lateral sclerosis in the early 2000s. According to these surveys, neurodegenerative diseases have received increased attention since 2010, which is consistent with the present findings [26,35]. This trend shows that the increased focus on these diseases as a central aspect of research is becoming more and more important.

### 4.1. Hot Spots and Possible Trends

During the review period, research on the KD focused on overweight and obesity, epilepsy, exercise, diabetes mellitus, and cancer. This is reflected in the most common keywords used in bibliometric analyses. These emphases result from the long-standing clinical application of the diet, particularly in epilepsy, and its influence on metabolism, weight regulation, and blood glucose levels, making it relevant to diseases such as diabetes and obesity. In addition, scientific trends in recent years have increasingly extended to other therapeutic areas, including cancer and neurodegenerative diseases [26,35,36,37]. The high interdisciplinary relevance and the proven metabolic effects make these topics central research priorities in the field of ketogenic nutrition. In addition to the studies described in the results section, the following section provides a brief description of the focal points mentioned in the context of current interest and the current state of research.

Studies of the KD in overweight and obesity have shown that significant weight loss can often be achieved. Discussed mechanisms include an inhibitory effect on appetite via satiety hormones and a direct effect of systemic ketone bodies through the KD [38]. In addition, obesity-related risk factors such as triglycerides, LDL, and HDL cholesterol may be positively influenced [39]. However, the weight-loss effect often wanes after six months, and effects beyond that are usually no greater than those observed in comparison groups [40,41,42].

With respect to Type 2 diabetes mellitus, there are other beneficial effects of the KD in addition to the often recommended weight loss. In addition to improvements in fasting insulin and blood glucose levels, studies have shown that a reduction in HbA1c levels can be achieved [40,43]. These effects appear to be superior to comparison groups such as the Mediterranean diet, high-protein diets, or moderate-carbohydrate diets [44]. Given the potential beneficial effects and the high global prevalence of obesity and diabetes mellitus, the interest in this area of research is not surprising.

Epilepsy is the oldest focus of ketogenic dietary therapies and continues to be intensively studied as a recognized therapeutic option. It is interesting to note that the anticonvulsant effect is not yet fully understood, although the basic approaches to this dietary therapy have been known since the 1920s [1,45]. This may explain why epilepsy has the second highest number of articles after overweight and obesity.

In the athletic setting, a KD has been shown to positively alter body composition. Loss of muscle mass can be minimized during weight loss on a KD [46,47]. Studies on performance improvement and strength gains show opposite effects. The KD shows more adverse or no different effects compared to high-carbohydrate diets [48,49,50]. It should be noted that the global fitness market has reached new heights with record membership in established markets such as the USA (23.7% of the population), UK (15.9%), Switzerland (14.9%), New Zealand (13.6%), and Germany (13.4%) [51,52]. At the same time, interest in the KD is growing, with an estimated market volume of up to USD 12.9 billion in 2024 and further growth potential through 2032 [53,54]. The increasing demand for exercise and healthy eating is indicative of a growing focus on health, making the KD more relevant as a complementary strategy for performance enhancement and weight management in the fitness sector.

The KD is being investigated as an adjunctive strategy in cancer therapy, particularly in the context of reduced glucose availability to tumor cells. Individual studies show possible benefits in terms of disease progression and survival time, but the results are often methodologically limited and inconclusive. It is also important to note the significant heterogeneity between different cancer types [55,56]. A particular problem with the KD is the often unintended significant weight loss, which is associated with a poorer prognosis for cancer patients [57,58,59]. In combination with chemotherapy, the side effects of the KD may pose additional risks. The already stressful loss of energy caused by therapy may be exacerbated by the diet, increasing the risk of malnutrition and cachexia [59,60]. This, in turn, can worsen the tolerability of cancer therapy and weaken the immune system, making infections and complications more likely [61]. Therefore, there is still a great deal of research to be conducted on the KD and its potential beneficial effects in cancer or in the context of chemotherapy.

### 4.2. Authors, Countries, and Journals

The publications included in this study come from 47 different countries. A comparison with other bibliometric reviews shows that the USA, England, Italy, Germany, China, Canada, and Japan are the ten most publishing countries [26,35,36,37]. This is roughly in line with the present survey. Among the authors, Jeff S. Volek stands out as the one who published the most on the KD during the period studied, which is consistent with the results of previous bibliometric analyses in which he was also one of the five most active researchers [26,35,37]. There is also some consistency in the journals published: *Nutrients*, *Epilepsy Research*, *PLoS One*, the *American Journal of Clinical Nutrition,* and *Nutrition* are among the top journals and are also found in different distributions among the most frequently represented journals of other bibliometric reviews [26,35,36,37].

### 4.3. Strengths and Limitations

In the present review, studies from the last five years dealing with the KD were specifically analyzed to obtain a current overview. A previous bibliometric analysis by Ye et al. [26], which included publications up to April 2022, showed a strong increase in publications in the period from 2019 to April 2022, so that a consideration of this period and the following years, despite the limitation to five years, can be seen as a useful update. In addition, the evaluation by Ye et al. was limited to the Web of Science database, whereas this analysis included the PubMed and ScienceDirect databases.

A strength of this data analysis is that, unlike other bibliometric studies, the data were manually extracted and analyzed from the publications using the principle of strict dual control, which allowed more data to be obtained, including the number of subjects and dropouts, and also reduced the limitations of the bibliometric software. One limitation of using bibliometric software is the listing of authors with different spellings or abbreviations.

A limitation of the analysis is that the search was limited to the PubMed and ScienceDirect databases. The bibliometric databases mentioned above were limited to the Web of Science or Web of Science Core Collection [26,36,37]. In this context, it should be noted that PubMed is one of the largest databases in the field of health-related sciences and, together with ScienceDirect, is one of the most frequently used databases for nutrition and medical research [62,63,64]. Accordingly, the databases with the highest relevance to the field of ketogenic nutrition were used. Another limitation is that the two most common topics, overweight/obesity and diabetes mellitus, are umbrella terms. For example, research on overweight and obesity includes studies that focus on metabolic syndrome, knee osteoarthritis, or appetite. Regarding diabetes mellitus, subdivisions include Type 1, Type 2, and prediabetes. Due to the large number of sub-topics and possible combinations of indications, these have not been considered individually in this paper but have been grouped under the respective general topic. However, a more detailed presentation would have led to a significant reduction in the frequency of mentions and thus to confusing results. However, there is also an opportunity to gain additional knowledge through further research. Another possible limitation is that the individual topics were not analyzed in depth. This has only been performed for the most common topics in the discussion section under “Hot spots and possible trends”. However, the aim of this paper is also to provide an overview of individual topics without going into depth. This has been achieved.

## 5. Conclusions

The analysis of research trends on the KD over the past five years demonstrates the importance and growing interest in this topic. The large number of studies, authors, journals, and indications found illustrates the broad consideration and intensive investigation of the KD. A large number of studies have been conducted in the last five years, particularly in the treatment of overweight and obesity. At the same time, it is clear that topics such as the KD for Alzheimer’s disease, kidney disease, and multiple sclerosis continue to be of interest and are the subject of ongoing research. Well-known topics such as epilepsy and cancer are also still the focus of research. Overall, the analysis shows that the KD remains an important and dynamic area of research that can continue to generate new knowledge and potential applications. There is still a high demand for research in this long-established, rediscovered field of research.

## Figures and Tables

**Table 1:** The top 10 topics in the field of ketogenic nutrition with ≥5 studies or publications in the databases PubMed and ScienceDirect from 2019 to August 2024.

Number	Subject Area	Frequency in All Studies
1	Overweight, obesity	56
2	Epilepsy	37
3	Sports	36
4	Diabetes mellitus	31
5	Cancer	28
6	Cardiovascular risk	21
7	Alzheimer’s disease, cognition	15
8	Safety, tolerability	8
9	Spinal cord and brain trauma	5
10	Migraine	5

**Table 2:** Total number of subjects, by sex (f/m), dropouts, and subjects remaining after dropout, also by sex (f/m), with number [n], mean, standard deviation [SD], median, minimum [min], and maximum [max].

	Study Data Available (N = 290)	Number	Mean Value	Median	Min.-Max.
**Total subjects**	290	151,856	523.64	37	5–125,982
Total subjects *	289	25,874	89.53	37	5–6369
**Female**	223	133,504	598.67	15	0–125,982
Female *	222	7522	33.88	15	0–518
**Male**	223	5958	26.72	14	0–247
**Total dropouts**	174	2728	15.61	5	0–229
**% Female retention after dropouts**	77		87.64	93.65	28.13–100
**% Male retention after dropouts**	71		87.28	100	30–100

* Excluding the data from Titcomb et al. [29] (n = 125.982 female subjects).

**Table 3:** The top 12 authors by number of publications in the field of the KD with ≥6 studies in the online literature databases PubMed and ScienceDirect from 2019 to August 2024.

Number	Author	Number of Articles	Number	Author	Number of Articles
**1**	Jeff S. Volek	14	7 *	Kim L. Bennell	6
**2**	Alex Buga	9	8 *	Pao-Hwa Lin	6
3 *	Christopher D. Crabtree	7	9 *	Rana S. Hinman	6
4 *	Madison L. Kackley	7	10 *	Stephen D. Phinney	6
**5**	Parker N. Hyde	7	11 *	Stephen J. Freedland	6
6 *	Ignacio Sajoux	6	12 *	Teryn N. Sapper	6

* The same number of articles. Random numbering of the authors.

**Table 4:** The top 10 countries of authors by the number of publications on the KD from 2019 to 2024 in the databases PubMed and ScienceDirect.

Number	Country	Frequency in All Studies
**1**	USA	562
**2**	Italy	175
**3**	United Kingdom	169
**4**	Germany	117
**5**	China	113
**6**	Australia	94
**7**	Canada	74
**8**	Japan	59
**9**	The Netherlands	51
**10**	Denmark	48

**Table 5:** The top 13 journals with ≥4 publications on the KD in the online literature databases PubMed and ScienceDirect from 2019 to 2024, indicating the most recent impact factor of the journal websites from 2023.

Number	Journal	Number of Articles	Impact Factor (2023)
**1**	*Nutrients*	47	4.8
**2**	*Clinical Nutrition*	10	6.6
**3**	*BMC Trials*	9	2.0
**4**	*Nutrition*	8	3.2
**5**	*The American Journal of Clinical Nutrition*	6	6.5
6 *	*The Journal of the International Society of Sports Nutrition*	5	4.5
6 *	*PLOS ONE*	5	2.9
6 *	*The Journal of Nutrition*	5	3.7
9 *	*BMC Musculoskeletal Disorders*	4	2.2
9 *	*Epilepsy Research*	4	2.0
9 *	*The International Journal of Environmental Research and Public Health*	4	4.6 **
9 *	*The Journal of Inherited Metabolic Diseases*	4	4.2
9 *	*Obesity*	4	4.2

* The same number of articles. Random numbering of the authors ** Last indication from 2021.

**Table 6:** The top 5 articles cited in ≥100 publications on the KD from 2019 to 2024 in the online literature databases PubMed and ScienceDirect.

Number	Title	First Author	Study Design	Journal	Year	Citations
**1**	Effects of 30 days of ketogenic diet on body composition, muscle strength, muscle area, metabolism, and performance in semi-professional soccer players	Paoli AA, et al. [30]	RCT	*The Journal of the International Society of Sports Nutrition*	2021	153
**2**	Ketonuria and Seizure Control in the Medium Chain Triglyceride and Classic Ketogenic Diets	Lowe H, et al. [31]	Observational study	*The Canadian Journal of Neurological Sciences*	2022	121
**3**	Effects of a medium-chain triglyceride-based ketogenic formula on cognitive function in patients with mild-to-moderate Alzheimer’s disease	Ota M, et al. [32]	CCT	*Neuroscience Letters*	2019	110
**4**	Effects of Ketogenic metabolic therapy on patients with breast cancer: A randomized controlled clinical trial	Khodabakhshi A, et al. [33]	RCT	*Clinical Nutrition*	2021	107
**5**	Association between ß-Hydroxybutyrate Plasma Concentrations after Hypocaloric Ketogenic Diets and Changes in Body Composition	Martins C, et al. [34]	CT	*The Journal of Nutrition*	2023	104

**Table 7:** The top 13 keywords mentioned in ≥10 publications on the KD from 2019 to 2024 in the databases PubMed and ScienceDirect.

Number	Keyword	Number of Articles
**1**	Ketogenic diet	91
**2**	Obesity	32
**3**	Ketosis	27
**4**	Diet	21
**5**	Weight loss	19
**6**	Ketone bodies	16
**7**	Exercise	15
8 *	Body composition	12
8 *	Epilepsy	12
8 *	Ketones	12
8 *	Nutrition	12
**12**	Metabolism	11
**13**	Low-carbohydrate diet	10

* The same number of articles. Random numbering of the authors.

## Data Availability

Not applicable.

## Supplementary Materials

The following supporting information can be downloaded at https://www.mdpi.com/article/10.3390/nu17091478/s1, Supplement S1: References of the 290 included studies.

## Abbreviations

The following abbreviations are used in this manuscript:

AcAc	Acetoacetate
CCT	Controlled clinical trials
CT	Clinical trials
KD	Ketogenic diet
LGIT	Low glycemic index therapy
MAD	Modified Atkins diet
MCTD	Medium-chain triglyceride diet
RCT	Randomized controlled trials
ßHB	β-hydroxybutyrate

## Appendix A

**Table A1:** Search strategy, including database, search term, and filter.

Database	Search Term	Filter
PubMed	“ketogenic” OR “modified Atkins” OR “Diet, Carbohydrate-Restricted” [Mesh] OR “low carb” OR “low-carbohydrate*” OR “medium-chain fatty acid*” OR “medium-chain triglyceride*”	Clinical Study, Clinical Trial, Clinical Trial, Phase I, Clinical Trial, Phase II, Clinical Trial, Phase III, Clinical Trial, Phase IV, Controlled Clinical Trial, Observational Study, Randomized Controlled Trial, from 2019 to 2024
ScienceDirect	“ketogenic” OR “low carb” OR “low-carbohydrate” OR “medium-chain fatty acid” OR “mct”	Clinical Study, Clinical Trial, Research Article, NOT Review, 2019–2024
