# Supplementary material for: Database Analysis of Application Areas and Global Trends in Ketogenic Diets from 2019 to 2024
Source: Nutrients. 2025 Apr 27;17(9):1478. doi: 10.3390/nu17091478 (PMC12073691; doi:10.3390/nu17091478)
Supplement: Supplementary file 1 [file nutrients-17-01478-s001.zip › nutrients-3591780-supplementary.pdf]

Supplement S1 - References of the 290 included studies

1. Ahmad, A.; Isherwood, C.; Umpleby, M.; Griffin, B. Effects of High and Low Sugar Diets on Cardiovascular Disease Risk Factors. *J. Nutr. Sci. Vitaminol. (Tokyo)* **2020**, *66*, S18-S24, doi:10.3177/jnsv.66.S18.
2. Akiyama, M.; Akiyama, T.; Saigusa, D.; Hishinuma, E.; Matsukawa, N.; Shibata, T.; Tsuchiya, H.; Mori, A.; Fujii, Y.; Mogami, Y.; et al. Comprehensive study of metabolic changes induced by a ketogenic diet therapy using GC/MS- and LC/MS-based metabolomics. *Seizure* **2023**, *107*, 52–59, doi:10.1016/j.seizure.2023.03.014.
3. Albanese, A.; Prevedello, L.; Markovich, M.; Busetto, L.; Vettor, R.; Foletto, M. Pre-operative Very Low Calorie Ketogenic Diet (VLCKD) vs. Very Low Calorie Diet (VLCD): Surgical Impact. *Obes. Surg.* **2019**, *29*, 292–296, doi:10.1007/s11695-018-3523-2.
4. Allan, N.P.; Yamamoto, B.Y.; Kunihiro, B.P.; Nunokawa, C.K.L.; Rubas, N.C.; Wells, R.K.; Umeda, L.; Phankitnirundorn, K.; Torres, A.; Peres, R.; et al. Ketogenic Diet Induced Shifts in the Gut Microbiome Associate with Changes to Inflammatory Cytokines and Brain-Related miRNAs in Children with Autism Spectrum Disorder. *Nutrients* **2024**, *16*, doi:10.3390/nu16101401.
5. Almodallal, Y.; Cook, K.; Lammert, L.M.; Lee, M.; Le-Rademacher, J.G.; Jatoi, A. Can older patients adopt and maintain a ketogenic diet? An observational study in support of clinical trials in older patients. *Medicine (Baltimore)* **2021**, *100*, e28033, doi:10.1097/MD.0000000000028033.
6. Al-Ozairi, E.; Reem, A.A.; El Samad, A.; Taghadom, E.; Al-Kandari, J.; Abdul-Ghani, M.; Oliver, N.; Whitcher, B.; Guess, N. A randomised crossover trial: Exploring the dose-response effect of carbohydrate restriction on glycaemia in people with well-controlled type 2 diabetes. *J. Hum. Nutr. Diet.* **2023**, *36*, 51–61, doi:10.1111/jhn.13030.
7. Anguah, K.O.-B.; Syed-Abdul, M.M.; Hu, Q.; Jacome-Sosa, M.; Heimowitz, C.; Cox, V.; Parks, E.J. Changes in Food Cravings and Eating Behavior after a Dietary Carbohydrate Restriction Intervention Trial. *Nutrients* **2019**, *12*, doi:10.3390/nu12010052.
8. Antonio Paoli, A.; Mancin, L.; Caprio, M.; Monti, E.; Narici, M.V.; Cenci, L.; Piccini, F.; Pincella, M.; Grigoletto, D.; Marcolin, G. Effects of 30 days of ketogenic diet on body composition, muscle strength, muscle area, metabolism, and performance in semi-professional soccer players. *J. Int. Soc. Sports Nutr.* **2021**, *18*, 62, doi:10.1186/s12970-021-00459-9.
9. Archna; Garg, D.; Goel, S.; Mukherjee, S.B.; Pemde, H.K.; Jain, P.; Sharma, S. Modified Atkins diet versus levetiracetam for non-surgical drug-resistant epilepsy in children: A randomized open-label study. *Seizure* **2022**, *103*, 61–67, doi:10.1016/j.seizure.2022.10.015.
10. Armeno, M.; Caballero, E.; Verini, A.; Reyes, G.; Galarza, N.; Cresta, A.; Caraballo, R.H. Telemedicine-versus outpatient-based initiation and management of ketogenic diet therapy in children with drug-resistant epilepsy during the COVID-19 pandemic. *Seizure* **2022**, *98*, 37–43, doi:10.1016/j.seizure.2022.03.023.
11. Arora, N.; Litofsky, N.S.; Golzy, M.; Aneja, R.; Staudenmyer, D.; Qualls, K.; Patil, S. Phase I single center trial of ketogenic diet for adults with traumatic brain injury. *Clin. Nutr. ESPEN* **2022**, *47*, 339–345, doi:10.1016/j.clnesp.2021.11.015.
12. Ashton, J.S.; Roberts, J.W.; Wakefield, C.J.; Page, R.M.; MacLaren, D.P.M.; Marwood, S.; Malone, J.J. The effects of medium chain triglyceride (MCT) supplementation using a C8:C10 ratio of 30:70 on cognitive performance in healthy young adults. *Physiol. Behav.* **2021**, *229*, 113252, doi:10.1016/j.physbeh.2020.113252.
13. Athinarayanan, S.J.; Hallberg, S.J.; McKenzie, A.L.; Lechner, K.; King, S.; McCarter, J.P.; Volek, J.S.; Phinney, S.D.; Krauss, R.M. Impact of a 2-year trial of nutritional ketosis on indices of cardiovascular disease risk in patients with type 2 diabetes. *Cardiovasc. Diabetol.* **2020**, *19*, 208, doi:10.1186/s12933-020-01178-2.
14. Augustus, E.; Granderson, I.; Rocke, K.D. The Impact of a Ketogenic Dietary Intervention on the Quality of Life of Stage II and III Cancer Patients: A Randomized Controlled Trial in the Caribbean. *Nutr. Cancer* **2021**, *73*, 1590–1600, doi:10.1080/01635581.2020.1803930.
15. Baart, A.M.; Schaminee, H.; Mensink, M.; Terink, R. Effect of a low carbohydrate, high fat diet versus a high carbohydrate diet on exercise efficiency and economy in recreational male athletes. *J. Sports Med. Phys. Fitness* **2023**, *63*, 282–291, doi:10.23736/S0022-4707.22.14066-1.
16. Bahr, L.S.; Bock, M.; Liebscher, D.; Bellmann-Strobl, J.; Franz, L.; Prüß, A.; Schumann, D.; Piper, S.K.; Kessler, C.S.; Steckhan, N.; et al. Ketogenic diet and fasting diet as Nutritional Approaches in Multiple

Sclerosis (NAMS): protocol of a randomized controlled study. *Trials* **2020**, *21*, 3, doi:10.1186/s13063-019-3928-9.

17. Barrea, L.; Muscogiuri, G.; Aprano, S.; Vetrani, C.; Alteriis, G. de; Varcamonti, L.; Verde, L.; Colao, A.; Savastano, S. Phase angle as an easy diagnostic tool for the nutritionist in the evaluation of inflammatory changes during the active stage of a very low-calorie ketogenic diet. *Int. J. Obes. (Lond)* **2022**, *46*, 1591–1597, doi:10.1038/s41366-022-01152-w.
18. Basciani, S.; Camajani, E.; Contini, S.; Persichetti, A.; Risi, R.; Bertoldi, L.; Strigari, L.; Prossomariti, G.; Watanabe, M.; Mariani, S.; et al. Very-Low-Calorie Ketogenic Diets With Whey, Vegetable, or Animal Protein in Patients With Obesity: A Randomized Pilot Study. *J. Clin. Endocrinol. Metab.* **2020**, *105*, doi:10.1210/clinem/dgaa336.
19. Battezzati, A.; Foppiani, A.; Leone, A.; Amicis, R. de; Spadafranca, A.; Mari, A.; Bertoli, S. Acute Insulin Secretory Effects of a Classic Ketogenic Meal in Healthy Subjects: A Randomized Cross-Over Study. *Nutrients* **2023**, *15*, doi:10.3390/nu15051119.
20. Belany, P.; Kackley, M.L.; Zhao, S.; Kluwe, B.; Buga, A.; Crabtree, C.D.; Nedungadi, D.; Kline, D.; Brock, G.; Simonetti, O.P.; et al. Effects of Hypocaloric Low-Fat, Ketogenic, and Ketogenic and Ketone Supplement Diets on Aldosterone and Renin. *J. Clin. Endocrinol. Metab.* **2023**, *108*, 1727–1739, doi:10.1210/clinem/dgad009.
21. Bennell, K.L.; Jones, S.E.; Hinman, R.S.; McManus, F.; Lamb, K.E.; Quicke, J.G.; Sumithran, P.; Prendergast, J.; George, E.S.; Holden, M.A.; et al. Effectiveness of a telehealth physiotherapist-delivered intensive dietary weight loss program combined with exercise in people with knee osteoarthritis and overweight or obesity: study protocol for the POWER randomized controlled trial. *BMC Musculoskelet. Disord.* **2022**, *23*, 733, doi:10.1186/s12891-022-05685-z.
22. Bennell, K.L.; Keating, C.; Lawford, B.J.; Kimp, A.J.; Egerton, T.; Brown, C.; Kasza, J.; Spiers, L.; Proietto, J.; Sumithran, P.; et al. Better Knee, Better Me™: effectiveness of two scalable health care interventions supporting self-management for knee osteoarthritis - protocol for a randomized controlled trial. *BMC Musculoskelet. Disord.* **2020**, *21*, 160, doi:10.1186/s12891-020-3166-z.
23. Bennell, K.L.; Lawford, B.J.; Keating, C.; Brown, C.; Kasza, J.; Mackenzie, D.; Metcalf, B.; Kimp, A.J.; Egerton, T.; Spiers, L.; et al. Comparing Video-Based, Telehealth-Delivered Exercise and Weight Loss Programs With Online Education on Outcomes of Knee Osteoarthritis : A Randomized Trial. *Ann. Intern. Med.* **2022**, *175*, 198–209, doi:10.7326/M21-2388.
24. Berrington, A.; Schreck, K.C.; Barron, B.J.; Blair, L.; Lin, D.D.M.; Hartman, A.L.; Kossoff, E.; Easter, L.; Whitlow, C.T.; Jung, Y.; et al. Cerebral Ketones Detected by 3T MR Spectroscopy in Patients with High-Grade Glioma on an Atkins-Based Diet. *AJNR Am. J. Neuroradiol.* **2019**, *40*, 1908–1915, doi:10.3174/ajnr.A6287.
25. Bharmal, S.H.; Cho, J.; Alarcon Ramos, G.C.; Ko, J.; Cameron-Smith, D.; Petrov, M.S. Acute Nutritional Ketosis and Its Implications for Plasma Glucose and Glucoregulatory Peptides in Adults with Prediabetes: A Crossover Placebo-Controlled Randomized Trial. *J. Nutr.* **2021**, *151*, 921–929, doi:10.1093/jn/nxaa417.
26. Bjurulf, B.; Magnus, P.; Hallböök, T.; Strømme, P. Potassium citrate and metabolic acidosis in children with epilepsy on the ketogenic diet: a prospective controlled study. *Dev. Med. Child Neurol.* **2020**, *62*, 57–61, doi:10.1111/dmcn.14393.
27. Bleeker, J.C.; Visser, G.; Clarke, K.; Ferdinandusse, S.; Haan, F.H. de; Houtkooper, R.H.; IJlst, L.; Kok, I.L.; Langeveld, M.; van der Pol, W.L.; et al. Nutritional ketosis improves exercise metabolism in patients with very long-chain acyl-CoA dehydrogenase deficiency. *J. Inherit. Metab. Dis.* **2020**, *43*, 787–799, doi:10.1002/jimd.12217.
28. Bock, M.; Steffen, F.; Zipp, F.; Bittner, S. Impact of Dietary Intervention on Serum Neurofilament Light Chain in Multiple Sclerosis. *Neurol. Neuroimmunol. Neuroinflamm.* **2022**, *9*, doi:10.1212/NXI.0000000000001102.
29. Bohnen, J.L.B.; Wigstrom, T.P.; Griggs, A.M.; Roytman, S.; Paalanen, N.; Andrews, H.A.; Bohnen, N.I.; Franklin, J.J.H.; McInnis, M.G. Ketogenic-Mimicking Diet as a Therapeutic Modality for Bipolar Disorder: Biomechanistic Rationale and Protocol for a Pilot Clinical Trial. *Nutrients* **2023**, *15*, doi:10.3390/nu15133068.
30. Bolyard, M.L.; Graziano, C.M.; Fontaine, K.R.; Sayer, R.D.; Fisher, G.; Plaisance, E.P. Tolerability and Acceptability of an Exogenous Ketone Monoester and Ketone Monoester/Salt Formulation in Humans. *Nutrients* **2023**, *15*, doi:10.3390/nu15234876.

31. Brandt, J.; Buchholz, A.; Henry-Barron, B.; Vizthum, D.; Avramopoulos, D.; Cervenka, M.C. Preliminary Report on the Feasibility and Efficacy of the Modified Atkins Diet for Treatment of Mild Cognitive Impairment and Early Alzheimer's Disease. *J. Alzheimers. Dis.* **2019**, *68*, 969–981, doi:10.3233/JAD-180995.
32. Brenton, J.N.; Lehner-Gulotta, D.; Woolbright, E.; Banwell, B.; Bergqvist, A.G.C.; Chen, S.; Coleman, R.; Conaway, M.; Goldman, M.D. Phase II study of ketogenic diets in relapsing multiple sclerosis: safety, tolerability and potential clinical benefits. *J. Neurol. Neurosurg. Psychiatry* **2022**, *93*, 637–644, doi:10.1136/jnnp-2022-329074.
33. Bruci, A.; Tuccinardi, D.; Tozzi, R.; Balena, A.; Santucci, S.; Frontani, R.; Mariani, S.; Basciani, S.; Spera, G.; Gnessi, L.; et al. Very Low-Calorie Ketogenic Diet: A Safe and Effective Tool for Weight Loss in Patients With Obesity and Mild Kidney Failure. *Nutrients* **2020**, *12*, doi:10.3390/nu12020333.
34. Buga, A.; Crabtree, C.D.; Stoner, J.T.; Decker, D.D.; Robinson, B.T.; Kackley, M.L.; Sapper, T.N.; Buxton, J.D.; D'Agostino, D.P.; McClure, T.S.; et al. Metabolic and ruck performance effects of a novel, light-weight, energy-dense ketogenic bar. *Exp. Physiol.* **2023**, *108*, 715–727, doi:10.1113/EP091029.
35. Buga, A.; Kackley, M.L.; Crabtree, C.D.; Bedell, T.N.; Robinson, B.T.; Stoner, J.T.; Decker, D.D.; Hyde, P.N.; LaFountain, R.A.; Brownlow, M.L.; et al. Fasting and diurnal blood ketonemia and glycemia responses to a six-week, energy-controlled ketogenic diet, supplemented with racemic R/S-BHB salts. *Clin. Nutr. ESPEN* **2023**, *54*, 277–287, doi:10.1016/j.clnesp.2023.01.030.
36. Buga, A.; Welton, G.L.; Scott, K.E.; Atwell, A.D.; Haley, S.J.; Esbenshade, N.J.; Abraham, J.; Buxton, J.D.; Ault, D.L.; Raabe, A.S.; et al. The Effects of Carbohydrate versus Fat Restriction on Lipid Profiles in Highly Trained, Recreational Distance Runners: A Randomized, Cross-Over Trial. *Nutrients* **2022**, *14*, doi:10.3390/nu14061135.
37. Burén, J.; Ericsson, M.; Damasceno, N.R.T.; Sjödin, A. A Ketogenic Low-Carbohydrate High-Fat Diet Increases LDL Cholesterol in Healthy, Young, Normal-Weight Women: A Randomized Controlled Feeding Trial. *Nutrients* **2021**, *13*, doi:10.3390/nu13030814.
38. Burén, J.; Svensson, M.; Liv, P.; Sjödin, A. Effects of a Ketogenic Diet on Body Composition in Healthy, Young, Normal-Weight Women: A Randomized Controlled Feeding Trial. *Nutrients* **2024**, *16*, doi:10.3390/nu16132030.
39. Burke, L.M.; Sharma, A.P.; Heikura, I.A.; Forbes, S.F.; Holloway, M.; McKay, A.K.A.; Bone, J.L.; Leckey, J.J.; Welvaert, M.; Ross, M.L. Crisis of confidence averted: Impairment of exercise economy and performance in elite race walkers by ketogenic low carbohydrate, high fat (LCHF) diet is reproducible. *PLoS One* **2020**, *15*, e0234027, doi:10.1371/journal.pone.0234027.
40. Cai, L.; Yin, J.; Ma, X.; Mo, Y.; Li, C.; Lu, W.; Bao, Y.; Zhou, J.; Jia, W. Low-carbohydrate diets lead to greater weight loss and better glucose homeostasis than exercise: a randomized clinical trial. *Front. Med.* **2021**, *15*, 460–471, doi:10.1007/s11684-021-0861-6.
41. Calabrese, L.; Scolnick, B.; Zupec-Kania, B.; Beckwith, C.; Costello, K.; Frank, G.K.W. Ketogenic diet and ketamine infusion treatment to target chronic persistent eating disorder psychopathology in anorexia nervosa: a pilot study. *Eat. Weight Disord.* **2022**, *27*, 3751–3757, doi:10.1007/s40519-022-01455-x.
42. Caprio, M.; Moriconi, E.; Camajani, E.; Feraco, A.; Marzolla, V.; Vitiello, L.; Proietti, S.; Armani, A.; Gorini, S.; Mammi, C.; et al. Very-low-calorie ketogenic diet vs hypocaloric balanced diet in the prevention of high-frequency episodic migraine: the EMIKETO randomized, controlled trial. *J. Transl. Med.* **2023**, *21*, 692, doi:10.1186/s12967-023-04561-1.
43. Carson, R.P.; Herber, D.L.; Pan, Z.; Phibbs, F.; Key, A.P.; Gouelle, A.; Ergish, P.; Armour, E.A.; Patel, S.; Duis, J. Nutritional Formulation for Patients with Angelman Syndrome: A Randomized, Double-Blind, Placebo-Controlled Study of Exogenous Ketones. *J. Nutr.* **2021**, *151*, 3628–3636, doi:10.1093/jn/nxab284.
44. Castaldo, G.; Rastrelli, L.; Galdo, G.; Molettieri, P.; Rotondi Aufiero, F.; Cereda, E. Aggressive weight-loss program with a ketogenic induction phase for the treatment of chronic plaque psoriasis: A proof-of-concept, single-arm, open-label clinical trial. *Nutrition* **2020**, *74*, 110757, doi:10.1016/j.nut.2020.110757.
45. Chang, C.R.; Francois, M.E.; Little, J.P. Restricting carbohydrates at breakfast is sufficient to reduce 24-hour exposure to postprandial hyperglycemia and improve glycemic variability. *Am. J. Clin. Nutr.* **2019**, *109*, 1302–1309, doi:10.1093/ajcn/nqy261.
46. Chen, O.; Blonquist, T.M.; Mah, E.; Sanoshy, K.; Beckman, D.; Nieman, K.M.; Winters, B.L.; Anthony, J.C.; Verdin, E.; Newman, J.C.; et al. Tolerability and Safety of a Novel Ketogenic Ester, Bis-Hexanoyl (R)-1,3-Butanediol: A Randomized Controlled Trial in Healthy Adults. *Nutrients* **2021**, *13*, doi:10.3390/nu13062066.

47. Chi, J.-T.; Lin, P.-H.; Tolstikov, V.; Howard, L.; Chen, E.Y.; Bussberg, V.; Greenwood, B.; Narain, N.R.; Kiebish, M.A.; Freedland, S.J. Serum metabolomic analysis of men on a low-carbohydrate diet for biochemically recurrent prostate cancer reveals the potential role of ketogenesis to slow tumor growth: a secondary analysis of the CAPS2 diet trial. *Prostate Cancer Prostatic Dis.* **2022**, *25*, 770–777, doi:10.1038/s41391-022-00525-6.
48. Chi, J.-T.; Lin, P.-H.; Tolstikov, V.; Oyekunle, T.; Alvarado, G.C.G.; Ramirez-Torres, A.; Chen, E.Y.; Bussberg, V.; Chi, B.; Greenwood, B.; et al. The influence of low-carbohydrate diets on the metabolic response to androgen-deprivation therapy in prostate cancer. *Prostate* **2021**, *81*, 618–628, doi:10.1002/pros.24136.
49. Cho, W.; Jung, H.; Hong, S.; Yang, H. in; Park, D.-H.; Suh, S.-H.; Lee, D.H.; Choe, Y.-S.; Kim, J.Y.; Lee, W.; et al. The effect of a short-term ketogenic diet on exercise efficiency during graded exercise in healthy adults. *J. Int. Soc. Sports Nutr.* **2023**, *20*, 2264278, doi:10.1080/15502783.2023.2264278.
50. Choi, A.H.; Delgado, M.; Chen, K.Y.; Chung, S.T.; Courville, A.; Turner, S.A.; Yang, S.; Airaghi, K.; Dustin, I.; McGurrin, P.; et al. A randomized feasibility trial of medium chain triglyceride-supplemented ketogenic diet in people with Parkinson's disease. *BMC Neurol.* **2024**, *24*, 106, doi:10.1186/s12883-024-03603-5.
51. Chyra, M.; Swietochowska, E.; Gorska-Flak, K.; Dudzinska, M.; Oswiecimska, J. The effect of the ketogenic diet on leptin, chemerin and resistin levels in children with epilepsy. *Neuro Endocrinol. Lett.* **2021**, *42*, 489–499.
52. Cipryan, L.; Kosek, V.; García, C.J.; Dostal, T.; Bechynska, K.; Hajslova, J.; Hofmann, P. A lipidomic and metabolomic signature of a very low-carbohydrate high-fat diet and high-intensity interval training: an additional analysis of a randomized controlled clinical trial. *Metabolomics* **2023**, *20*, 10, doi:10.1007/s11306-023-02071-1.
53. Cohen, C.W.; Fontaine, K.R.; Arend, R.C.; Gower, B.A. A Ketogenic Diet Is Acceptable in Women with Ovarian and Endometrial Cancer and Has No Adverse Effects on Blood Lipids: A Randomized, Controlled Trial. *Nutr. Cancer* **2020**, *72*, 584–594, doi:10.1080/01635581.2019.1645864.
54. Correa, L.L.; Moretti, A.; Sousa, P.A.M. de; Dinis, L.; Souza, M.F. de; Tostes, I.; Nuñez-Garcia, M.; Sajoux, I. Effectiveness and Safety of a Very Low-Calorie Ketogenic Diet on Weight Regain Following Bariatric Surgery. *Obes. Surg.* **2021**, *31*, 5383–5390, doi:10.1007/s11695-021-05703-4.
55. Crabtree, C.D.; Blade, T.; Hyde, P.N.; Buga, A.; Kackley, M.L.; Sapper, T.N.; Panda, O.; Roa-Diaz, S.; Anthony, J.C.; Newman, J.C.; et al. Bis Hexanoyl (R)-1,3-Butanediol, a Novel Ketogenic Ester, Acutely Increases Circulating r- and s-ß-Hydroxybutyrate Concentrations in Healthy Adults. *J. Am. Nutr. Assoc.* **2023**, *42*, 169–177, doi:10.1080/07315724.2021.2015476.
56. Crabtree, C.D.; Kackley, M.L.; Buga, A.; Fell, B.; LaFountain, R.A.; Hyde, P.N.; Sapper, T.N.; Kraemer, W.J.; Scandling, D.; Simonetti, O.P.; et al. Comparison of Ketogenic Diets with and without Ketone Salts versus a Low-Fat Diet: Liver Fat Responses in Overweight Adults. *Nutrients* **2021**, *13*, doi:10.3390/nu13030966.
57. Cukoski, S.; Lindemann, C.H.; Arjune, S.; Todorova, P.; Brecht, T.; Kühn, A.; Oehm, S.; Strubl, S.; Becker, I.; Kämmerer, U.; et al. Feasibility and impact of ketogenic dietary interventions in polycystic kidney disease: KETO-ADPKD-a randomized controlled trial. *Cell Rep. Med.* **2023**, *4*, 101283, doi:10.1016/j.xcrm.2023.101283.
58. Cunha, G.M.; Correa de Mello, L.L.; Hasenstab, K.A.; Spina, L.; Bussade, I.; Prata Mesiano, J.M.; Coutinho, W.; Guzman, G.; Sajoux, I. MRI estimated changes in visceral adipose tissue and liver fat fraction in patients with obesity during a very low-calorie-ketogenic diet compared to a standard low-calorie diet. *Clin. Radiol.* **2020**, *75*, 526–532, doi:10.1016/j.crad.2020.02.014.
59. Cunha, G.M.; Guzman, G.; Correa De Mello, L.L.; Trein, B.; Spina, L.; Bussade, I.; Marques Prata, J.; Sajoux, I.; Countinho, W. Efficacy of a 2-Month Very Low-Calorie Ketogenic Diet (VLCKD) Compared to a Standard Low-Calorie Diet in Reducing Visceral and Liver Fat Accumulation in Patients With Obesity. *Front. Endocrinol. (Lausanne)* **2020**, *11*, 607, doi:10.3389/fendo.2020.00607.
60. D'Abbondanza, M.; Ministrini, S.; Pucci, G.; Nulli Migliola, E.; Martorelli, E.-E.; Gandolfo, V.; Siepi, D.; Lupattelli, G.; Vaudo, G. Very Low-Carbohydrate Ketogenic Diet for the Treatment of Severe Obesity and Associated Non-Alcoholic Fatty Liver Disease: The Role of Sex Differences. *Nutrients* **2020**, *12*, doi:10.3390/nu12092748.
61. Dahlin, M.; Singleton, S.S.; David, J.A.; Basuchoudhary, A.; Wickström, R.; Mazumder, R.; Prast-Nielsen, S. Higher levels of Bifidobacteria and tumor necrosis factor in children with drug-resistant epilepsy are associated with anti-seizure response to the ketogenic diet. *EBioMedicine* **2022**, *80*, 104061, doi:10.1016/j.ebiom.2022.104061.

62. Dellis, D.; Tsilingiris, D.; Eleftheriadou, I.; Tentolouris, A.; Sfikakis, P.P.; Dellis, G.; Karanasiou, M.; Meimari, A.; Dimosthenopoulos, C.; Lazarou, S.; et al. Carbohydrate restriction in the morning increases weight loss effect of a hypocaloric Mediterranean type diet: a randomized, parallel group dietary intervention in overweight and obese subjects. *Nutrition* **2020**, *71*, 110578, doi:10.1016/j.nut.2019.110578.
63. Demirel, A.; Li, J.; Morrow, C.; Barnes, S.; Jansen, J.; Gower, B.; Kirksey, K.; Redden, D.; Yarar-Fisher, C. Evaluation of a ketogenic diet for improvement of neurological recovery in individuals with acute spinal cord injury: study protocol for a randomized controlled trial. *Trials* **2020**, *21*, 372, doi:10.1186/s13063-020-04273-7.
64. Deru, L.S.; Gipson, E.Z.; Hales, K.E.; Bikman, B.T.; Davidson, L.E.; Horne, B.D.; LeCheminant, J.D.; Tucker, L.A.; Bailey, B.W. The Effects of a High-Carbohydrate versus a High-Fat Shake on Biomarkers of Metabolism and Glycemic Control When Used to Interrupt a 38-h Fast: A Randomized Crossover Study. *Nutrients* **2024**, *16*, doi:10.3390/nu16010164.
65. Di Lorenzo, C.; Pinto, A.; Ienca, R.; Coppola, G.; Sirianni, G.; Di Lorenzo, G.; Parisi, V.; Serrao, M.; Spagnoli, A.; Vestri, A.; et al. A Randomized Double-Blind, Cross-Over Trial of very Low-Calorie Diet in Overweight Migraine Patients: A Possible Role for Ketones? *Nutrients* **2019**, *11*, doi:10.3390/nu11081742.
66. Di Rosa, C.; Lattanzi, G.; Spiezia, C.; Imperia, E.; Piccirilli, S.; Beato, I.; Gaspa, G.; Micheli, V.; Joannon, F. de; Vallecorsa, N.; et al. Mediterranean Diet versus Very Low-Calorie Ketogenic Diet: Effects of Reaching 5% Body Weight Loss on Body Composition in Subjects with Overweight and with Obesity-A Cohort Study. *Int. J. Environ. Res. Public Health* **2022**, *19*, doi:10.3390/ijerph192013040.
67. Dorans, K.S.; Bazzano, L.A.; Qi, L.; He, H.; Appel, L.J.; Samet, J.M.; Chen, J.; Mills, K.T.; Nguyen, B.T.; O'Brien, M.J.; et al. Low-carbohydrate dietary pattern on glycemic outcomes trial (ADEPT) among individuals with elevated hemoglobin A1c: study protocol for a randomized controlled trial. *Trials* **2021**, *22*, 108, doi:10.1186/s13063-020-05001-x.
68. Dorans, K.S.; Bazzano, L.A.; Qi, L.; He, H.; Chen, J.; Appel, L.J.; Chen, C.-S.; Hsieh, M.-H.; Hu, F.B.; Mills, K.T.; et al. Effects of a Low-Carbohydrate Dietary Intervention on Hemoglobin A1c: A Randomized Clinical Trial. *JAMA Netw. Open* **2022**, *5*, e2238645, doi:10.1001/jamanetworkopen.2022.38645.
69. Dou, Y.; Jiang, Y.; Chen, X.; Zhang, Y.; Wang, Y.; Chen, H.; He, W.; Yan, W. Intermittent dietary carbohydrate restriction versus calorie restriction and cardiometabolic profiles: A randomized trial. *Obesity (Silver Spring)* **2023**, *31*, 2260–2271, doi:10.1002/oby.23855.
70. Draaisma, J.M.T.; Hampsink, B.M.; Janssen, M.; van Houdt, N.B.M.; Linders, E.T.A.M.; Willemsen, M.A. The Ketogenic Diet and Its Effect on Bone Mineral Density: A Retrospective Observational Cohort Study. *Neuropediatrics* **2019**, *50*, 353–358, doi:10.1055/s-0039-1693059.
71. Drabińska, N.; Romaszko, J.; White, P. The effect of isocaloric, energy-restrictive, KETOgenic diet on metabolism, inflammation, nutrition deficiencies and oxidative stress in women with overweight and obesity (KETO-MINOX): Study protocol. *PLoS One* **2023**, *18*, e0285283, doi:10.1371/journal.pone.0285283.
72. Dressler, A.; Benninger, F.; Trimmel-Schwahofer, P.; Gröppel, G.; Porsche, B.; Abraham, K.; Mühlebner, A.; Samueli, S.; Male, C.; Feucht, M. Efficacy and tolerability of the ketogenic diet versus high-dose adrenocorticotropic hormone for infantile spasms: A single-center parallel-cohort randomized controlled trial. *Epilepsia* **2019**, *60*, 441–451, doi:10.1111/epi.14679.
73. Du, Y.; Wang, J.; Li, S.; Dennis, B.; Meireles, C.; Siddiqui, N.; Patel, D.; Gelfond, J.; Li, C.; Faruqui, S.-H.-A.; et al. A technology assisted precision ketogenic diet intervention for cardio-renal-metabolic health in overweight or obese adults: Protocol for a randomized controlled trial. *Contemp. Clin. Trials* **2022**, *119*, 106845, doi:10.1016/j.cct.2022.106845.
74. Durkalec-Michalski, K.; Nowaczyk, P.M.; Główka, N.; Ziobrowska, A.; Podgórski, T. Is a Four-Week Ketogenic Diet an Effective Nutritional Strategy in CrossFit-Trained Female and Male Athletes? *Nutrients* **2021**, *13*, doi:10.3390/nu13030864.
75. Durkalec-Michalski, K.; Nowaczyk, P.M.; Siedzik, K. Effect of a four-week ketogenic diet on exercise metabolism in CrossFit-trained athletes. *J. Int. Soc. Sports Nutr.* **2019**, *16*, 16, doi:10.1186/s12970-019-0284-9.
76. Durrer, C.; McKelvey, S.; Singer, J.; Batterham, A.M.; Johnson, J.D.; Gudmundson, K.; Wortman, J.; Little, J.P. A randomized controlled trial of pharmacist-led therapeutic carbohydrate and energy restriction in type 2 diabetes. *Nat. Commun.* **2021**, *12*, 5367, doi:10.1038/s41467-021-25667-4.
77. Eiden, M.; Christinat, N.; Chakrabarti, A.; Sonnay, S.; Miroz, J.-P.; Cuenoud, B.; Oddo, M.; Masoodi, M. Discovery and validation of temporal patterns involved in human brain ketometabolism in cerebral

microdialysis fluids of traumatic brain injury patients. *EBioMedicine* **2019**, *44*, 607–617, doi:10.1016/j.ebiom.2019.05.054.
78. El-Shafie, A.M.; Bahbah, W.A.; Abd El Naby, S.A.; Omar, Z.A.; Basma, E.M.; Hegazy, A.A.A.; El Zefzaf, H.M.S. Impact of two ketogenic diet types in refractory childhood epilepsy. *Pediatr. Res.* **2023**, *94*, 1978–1989, doi:10.1038/s41390-023-02554-w.
79. Eneli, I.; Xu, J.; Tindall, A.; Watowicz, R.; Worthington, J.; Tanner, K.; Pratt, K.; Walston, M. Using a Revised Protein-Sparing Modified Fast (rPSMF) for Children and Adolescents with Severe Obesity: A Pilot Study. *Int. J. Environ. Res. Public Health* **2019**, *16*, doi:10.3390/ijerph16173061.
80. Falkenhain, K.; Locke, S.R.; Lowe, D.A.; Lee, T.; Singer, J.; Weiss, E.J.; Little, J.P. Use of an mHealth Ketogenic Diet App Intervention and User Behaviors Associated With Weight Loss in Adults With Overweight or Obesity: Secondary Analysis of a Randomized Clinical Trial. *JMIR Mhealth Uhealth* **2022**, *10*, e33940, doi:10.2196/33940.
81. Falkenhain, K.; Locke, S.R.; Lowe, D.A.; Reitsma, N.J.; Lee, T.; Singer, J.; Weiss, E.J.; Little, J.P. Keyto app and device versus WW app on weight loss and metabolic risk in adults with overweight or obesity: A randomized trial. *Obesity (Silver Spring)* **2021**, *29*, 1606–1614, doi:10.1002/oby.23242.
82. Fan, L.; Zhu, X.; Sun, S.; Yu, C.; Huang, X.; Ness, R.; Dugan, L.L.; Shu, L.; Seidner, D.L.; Murff, H.J.; et al. Ca:Mg ratio, medium-chain fatty acids, and the gut microbiome. *Clin. Nutr.* **2022**, *41*, 2490–2499, doi:10.1016/j.clnu.2022.08.031.
83. Field, R.; Pourkazemi, F.; Rooney, K. Effects of a Low-Carbohydrate Ketogenic Diet on Reported Pain, Blood Biomarkers and Quality of Life in Patients with Chronic Pain: A Pilot Randomized Clinical Trial. *Pain Med.* **2022**, *23*, 326–338, doi:10.1093/pm/pnab278.
84. Field, R.J.; Field, T.J.; Pourkazemi, F.; Rooney, K.B. Experience of participants with chronic pain in a pilot randomized clinical trial using a ketogenic diet. *Pain Manag.* **2022**, *12*, 313–322, doi:10.2217/pmt-2021-0084.
85. Folwaczny, A.; Waldmann, E.; Altenhofer, J.; Henze, K.; Parhofer, K.G. Postprandial Lipid Metabolism in Normolipidemic Subjects and Patients with Mild to Moderate Hypertriglyceridemia: Effects of Test Meals Containing Saturated Fatty Acids, Mono-Unsaturated Fatty Acids, or Medium-Chain Fatty Acids. *Nutrients* **2021**, *13*, doi:10.3390/nu13051737.
86. Formisano, E.; Schiavetti, I.; Gradaschi, R.; Gardella, P.; Romeo, C.; Pisciotta, L.; Sukkar, S.G. The Real-Life Use of a Protein-Sparing Modified Fast Diet by Nasogastric Tube (ProMoFasT) in Adults with Obesity: An Open-Label Randomized Controlled Trial. *Nutrients* **2023**, *15*, doi:10.3390/nu15224822.
87. Fortier, M.; Castellano, C.-A.; St-Pierre, V.; Myette-Côté, É.; Langlois, F.; Roy, M.; Morin, M.-C.; Bocti, C.; Fulop, T.; Godin, J.-P.; et al. A ketogenic drink improves cognition in mild cognitive impairment: Results of a 6-month RCT. *Alzheimers. Dement.* **2021**, *17*, 543–552, doi:10.1002/alz.12206.
88. Freedland, S.J.; Allen, J.; Jarman, A.; Oyekunle, T.; Armstrong, A.J.; Moul, J.W.; Sandler, H.M.; Posadas, E.; Levin, D.; Wiggins, E.; et al. A Randomized Controlled Trial of a 6-Month Low-Carbohydrate Intervention on Disease Progression in Men with Recurrent Prostate Cancer: Carbohydrate and Prostate Study 2 (CAPS2). *Clin. Cancer Res.* **2020**, *26*, 3035–3043, doi:10.1158/1078-0432.CCR-19-3873.
89. Freedland, S.J.; Howard, L.; Allen, J.; Smith, J.; Stout, J.; Aronson, W.; Inman, B.A.; Armstrong, A.J.; George, D.; Westman, E.; et al. A lifestyle intervention of weight loss via a low-carbohydrate diet plus walking to reduce metabolic disturbances caused by androgen deprivation therapy among prostate cancer patients: carbohydrate and prostate study 1 (CAPS1) randomized controlled trial. *Prostate Cancer Prostatic Dis.* **2019**, *22*, 428–437, doi:10.1038/s41391-019-0126-5.
90. Furuta, Y.; Manita, D.; Hirowatari, Y.; Shoji, K.; Ogata, H.; Tanaka, A.; Kawabata, T. Postprandial fatty acid metabolism with coconut oil in young females: a randomized, single-blind, crossover trial. *Am. J. Clin. Nutr.* **2023**, *117*, 1240–1247, doi:10.1016/j.ajcnut.2023.03.015.
91. Gao, M.; Kirk, M.; Lash, E.; Knight, H.; Michalopoulou, M.; Guess, N.; Browning, M.; Weich, S.; Burnet, P.; Jebb, S.A.; et al. Evaluating the efficacy and mechanisms of a ketogenic diet as adjunctive treatment for people with treatment-resistant depression: A protocol for a randomised controlled trial. *J. Psychiatr. Res.* **2024**, *174*, 230–236, doi:10.1016/j.jpsychires.2024.04.023.
92. Gardner, C.D.; Landry, M.J.; Perelman, D.; Petlura, C.; Durand, L.R.; Aronica, L.; Crimarco, A.; Cunanan, K.M.; Chang, A.; Dant, C.C.; et al. Effect of a ketogenic diet versus Mediterranean diet on glycated hemoglobin in individuals with prediabetes and type 2 diabetes mellitus: The interventional Keto-Med randomized crossover trial. *Am. J. Clin. Nutr.* **2022**, *116*, 640–652, doi:10.1093/ajcn/nqac154.

93. Gepner, Y.; Shelef, I.; Komy, O.; Cohen, N.; Schwarzfuchs, D.; Bril, N.; Rein, M.; Serfaty, D.; Kenigsbuch, S.; Zelicha, H.; et al. The beneficial effects of Mediterranean diet over low-fat diet may be mediated by decreasing hepatic fat content. *J. Hepatol.* **2019**, *71*, 379–388, doi:10.1016/j.jhep.2019.04.013.
94. Ghorbanian, B.; Wong, A.; Iranpour, A. The effect of dietary carbohydrate restriction and aerobic exercise on retinol binding protein 4 (RBP4) and fatty acid binding protein 5 (FABP5) in middle-aged men with metabolic syndrome. *Br. J. Nutr.* **2023**, *130*, 553–563, doi:10.1017/S0007114522003580.
95. Goldenshluger, A.; Constantini, K.; Goldstein, N.; Shelef, I.; Schwarzfuchs, D.; Zelicha, H.; Yaskolka Meir, A.; Tsaban, G.; Chassidim, Y.; Gepner, Y. Effect of Dietary Strategies on Respiratory Quotient and Its Association with Clinical Parameters and Organ Fat Loss: A Randomized Controlled Trial. *Nutrients* **2021**, *13*, doi:10.3390/nu13072230.
96. Graybeal, A.J.; Kreutzer, A.; Moss, K.; Rack, P.; Augsburger, G.; Braun-Trocchio, R.; Willis, J.L.; Shah, M. Chronic and Postprandial Metabolic Responses to a Ketogenic Diet Compared to High-Carbohydrate and Habitual Diets in Trained Competitive Cyclists and Triathletes: A Randomized Crossover Trial. *Int. J. Environ. Res. Public Health* **2023**, *20*, doi:10.3390/ijerph20021110.
97. Graybeal, A.J.; Kreutzer, A.; Rack, P.; Moss, K.; Augsburger, G.; Willis, J.L.; Braun-Trocchio, R.; Shah, M. Perceptions of appetite do not match hormonal measures of appetite in trained competitive cyclists and triathletes following a ketogenic diet compared to a high-carbohydrate or habitual diet: A randomized crossover trial. *Nutr. Res.* **2021**, *93*, 111–123, doi:10.1016/j.nutres.2021.07.008.
98. Griauzde, D.H.; O'Brien, A.; Yancy, W.S.; Richardson, C.R.; Krinock, J.; DeJonckheere, M.; Isaman, D.J.M.; Vanias, K.; Shopinski, S.; Saslow, L.R. Testing a very low-carbohydrate adaption of the Diabetes Prevention Program among adults with prediabetes: study protocol for the Lifestyle Education about prediabetes (LEAP) trial. *Trials* **2022**, *23*, 827, doi:10.1186/s13063-022-06770-3.
99. Gross, E.; Putananickal, N.; Orsini, A.-L.; Schmidt, S.; Vogt, D.R.; Cichon, S.; Sandor, P.; Fischer, D. Efficacy and safety of exogenous ketone bodies for preventive treatment of migraine: A study protocol for a single-centred, randomised, placebo-controlled, double-blind crossover trial. *Trials* **2019**, *20*, 61, doi:10.1186/s13063-018-3120-7.
100. Guevara-Cruz, M.; Hernández-Gómez, K.G.; Condado-Huerta, C.; González-Salazar, L.E.; Peña-Flores, A.K.; Pichardo-Ontiveros, E.; Serralde-Zúñiga, A.E.; Sánchez-Tapia, M.; Maya, O.; Medina-Vera, I.; et al. Intermittent fasting, calorie restriction, and a ketogenic diet improve mitochondrial function by reducing lipopolysaccharide signaling in monocytes during obesity: A randomized clinical trial. *Clin. Nutr.* **2024**, *43*, 1914–1928, doi:10.1016/j.clnu.2024.06.036.
101. Gupta, S.; Dabla, S.; Kaushik, J.S. Modified Atkins Diet vs Low Glycemic Index Treatment for Drug-Resistant Epilepsy in Children: An Open Label, Randomized Controlled Trial. *Indian Pediatr.* **2021**, *58*, 815–819.
102. Gutiérrez-Repiso, C.; Hernández-García, C.; García-Almeida, J.M.; Bellido, D.; Martín-Núñez, G.M.; Sánchez-Alcoholado, L.; Alcaide-Torres, J.; Sajoux, I.; Tinahones, F.J.; Moreno-Indias, I. Effect of Synbiotic Supplementation in a Very-Low-Calorie Ketogenic Diet on Weight Loss Achievement and Gut Microbiota: A Randomized Controlled Pilot Study. *Mol. Nutr. Food Res.* **2019**, *63*, e1900167, doi:10.1002/mnfr.201900167.
103. Guzel, O.; Uysal, U.; Arslan, N. Efficacy and tolerability of olive oil-based ketogenic diet in children with drug-resistant epilepsy: A single center experience from Turkey. *Eur. J. Paediatr. Neurol.* **2019**, *23*, 143–151, doi:10.1016/j.ejpn.2018.11.007.
104. Guzmán, G.; Sajoux, I.; Aller, R.; Izaola, O.; Luis, D. de. Metodología multidisciplinar y dieta cetogénica en la práctica clínica real: eficacia y rapidez en la pérdida de peso. Análisis de supervivencia. Estudio PROMET Lipoinflamación. *Nutr. Hosp.* **2020**, *34*, 497–505, doi:10.20960/nh.02997.
105. Hägele, F.A.; Dörner, R.; Koop, J.; Lübken, M.; Seidel, U.; Rimbach, G.; Müller, M.J.; Bosy-Westphal, A. Impact of one-day fasting, ketogenic diet or exogenous ketones on control of energy balance in healthy participants. *Clin. Nutr. ESPEN* **2023**, *55*, 292–299, doi:10.1016/j.clnesp.2023.03.025.
106. Hall, K.D.; Guo, J.; Courville, A.B.; Boring, J.; Brychta, R.; Chen, K.Y.; Darcey, V.; Forde, C.G.; Gharib, A.M.; Gallagher, I.; et al. Effect of a plant-based, low-fat diet versus an animal-based, ketogenic diet on ad libitum energy intake. *Nat. Med.* **2021**, *27*, 344–353, doi:10.1038/s41591-020-01209-1.
107. Hall, M.; Hinman, R.S.; Knox, G.; Spiers, L.; Sumithran, P.; Murphy, N.J.; McManus, F.; Lamb, K.E.; Cicuittini, F.; Hunter, D.J.; et al. Effects of adding a diet intervention to exercise on hip osteoarthritis pain: protocol for the ECHO randomized controlled trial. *BMC Musculoskelet. Disord.* **2022**, *23*, 215, doi:10.1186/s12891-022-05128-9.

108. Han, Y.; Cheng, B.; Guo, Y.; Wang, Q.; Yang, N.; Lin, P. A Low-Carbohydrate Diet Realizes Medication Withdrawal: A Possible Opportunity for Effective Glycemic Control. *Front. Endocrinol. (Lausanne)* **2021**, *12*, 779636, doi:10.3389/fendo.2021.779636.
109. Harris, A.; Hinman, R.S.; Lawford, B.J.; Egerton, T.; Keating, C.; Brown, C.; Metcalf, B.; Spiers, L.; Sumithran, P.; Quicke, J.G.; et al. Cost-Effectiveness of Telehealth-Delivered Exercise and Dietary Weight Loss Programs for Knee Osteoarthritis Within a Twelve-Month Randomized Trial. *Arthritis Care Res. (Hoboken)* **2023**, *75*, 1311–1319, doi:10.1002/acr.25022.
110. Harvey, C.J.D.C.; Schofield, G.M.; Zinn, C.; Thornley, S. Effects of differing levels of carbohydrate restriction on mood achievement of nutritional ketosis, and symptoms of carbohydrate withdrawal in healthy adults: A randomized clinical trial. *Nutrition* **2019**, *67-68S*, 100005, doi:10.1016/j.nutx.2019.100005.
111. Heidt, C.; Pons-Kühnemann, J.; Kämmerer, U.; Marquardt, T.; Reuss-Borst, M. MCT-Induced Ketosis and Fiber in Rheumatoid Arthritis (MIKARA)-Study Protocol and Primary Endpoint Results of the Double-Blind Randomized Controlled Intervention Study Indicating Effects on Disease Activity in RA Patients. *Nutrients* **2023**, *15*, doi:10.3390/nu15173719.
112. Henderson, L.R.; van den Berg, M.; Shaw, D.M. The effect of a 2 week ketogenic diet, versus a carbohydrate-based diet, on cognitive performance, mood and subjective sleepiness during 36 h of extended wakefulness in military personnel: An exploratory study. *J. Sleep Res.* **2023**, *32*, e13832, doi:10.1111/jsr.13832.
113. Hengist, A.; Davies, R.G.; Rogers, P.J.; Brunstrom, J.M.; van Loon, L.J.C.; Walhin, J.-P.; Thompson, D.; Koumanov, F.; Betts, J.A.; Gonzalez, J.T. Restricting sugar or carbohydrate intake does not impact physical activity level or energy intake over 24 h despite changes in substrate use: a randomised crossover study in healthy men and women. *Eur. J. Nutr.* **2023**, *62*, 921–940, doi:10.1007/s00394-022-03048-x.
114. Herber, D.L.; Weeber, E.J.; D'Agostino, D.P.; Duis, J. Evaluation of the safety and tolerability of a nutritional Formulation in patients with ANgelman Syndrome (FANS): study protocol for a randomized controlled trial. *Trials* **2020**, *21*, 60, doi:10.1186/s13063-019-3996-x.
115. Hoogeveen, I.J.; Boer, F. de; Boonstra, W.F.; van der Schaaf, C.J.; Steuerwald, U.; Sibeijn-Kuiper, A.J.; Vegter, R.J.K.; van der Hoeven, J.H.; Heiner-Fokkema, M.R.; Clarke, K.C.; et al. Effects of acute nutritional ketosis during exercise in adults with glycogen storage disease type IIIa are phenotype-specific: An investigator-initiated, randomized, crossover study. *J. Inherit. Metab. Dis.* **2021**, *44*, 226–239, doi:10.1002/jimd.12302.
116. Hsu, C.; Huang, Y.-W.; Lin, S.-M.; Lu, C.-S.; Chen, C.-Y.; Chang, C.-K. Low- or moderate-carbohydrate calorie-restricted diets have similar effects on body composition and taekwondo performance after high-carbohydrate recovery meals. *Eur. J. Sport Sci.* **2023**, *23*, 1983–1992, doi:10.1080/17461391.2023.2199423.
117. Hwang, C.-L.; Ranieri, C.; Szczurek, M.R.; Ellythy, A.M.; Elokda, A.; Mahmoud, A.M.; Phillips, S.A. The Effect of Low-Carbohydrate Diet on Macrovascular and Microvascular Endothelial Function is Not Affected by the Provision of Caloric Restriction in Women with Obesity: A Randomized Study. *Nutrients* **2020**, *12*, doi:10.3390/nu12061649.
118. Hyde, P.N.; Sapper, T.N.; Crabtree, C.D.; LaFountain, R.A.; Bowling, M.L.; Buga, A.; Fell, B.; McSwiney, F.T.; Dickerson, R.M.; Miller, V.J.; et al. Dietary carbohydrate restriction improves metabolic syndrome independent of weight loss. *JCI Insight* **2019**, *4*, doi:10.1172/jci.insight.128308.
119. Hyde, P.N.; Sapper, T.N.; LaFountain, R.A.; Kackley, M.L.; Buga, A.; Fell, B.; Crabtree, C.D.; Phinney, S.D.; Miller, V.J.; King, S.M.; et al. Effects of Palm Stearin versus Butter in the Context of Low-Carbohydrate/High-Fat and High-Carbohydrate/Low-Fat Diets on Circulating Lipids in a Controlled Feeding Study in Healthy Humans. *Nutrients* **2021**, *13*, doi:10.3390/nu13061944.
120. Iacovides, S.; Goble, D.; Paterson, B.; Meiring, R.M. Three consecutive weeks of nutritional ketosis has no effect on cognitive function, sleep, and mood compared with a high-carbohydrate, low-fat diet in healthy individuals: a randomized, crossover, controlled trial. *Am. J. Clin. Nutr.* **2019**, *110*, 349–357, doi:10.1093/ajcn/nqz073.
121. Iacovides, S.; Maloney, S.K.; Bhana, S.; Angamia, Z.; Meiring, R.M. Could the ketogenic diet induce a shift in thyroid function and support a metabolic advantage in healthy participants? A pilot randomized-controlled-crossover trial. *PLoS One* **2022**, *17*, e0269440, doi:10.1371/journal.pone.0269440.
122. Iyikesici, M.S. Langzeitüberlebens-Outcomes der metabolisch unterstützten Chemotherapie mit Gemcitabin oder FOLFIRINOX in Kombination mit ketogener Ernährung, Hyperthermie und hyperbarer Sauerstofftherapie beim metastasierenden Pankreaskarzinom. *Complement. Med. Res.* **2020**, *27*, 31–39, doi:10.1159/000502135.

123. Jansen, L.T.; Yang, N.; Wong, J.M.W.; Mehta, T.; Allison, D.B.; Ludwig, D.S.; Ebbeling, C.B. Prolonged Glycemic Adaptation Following Transition From a Low- to High-Carbohydrate Diet: A Randomized Controlled Feeding Trial. *Diabetes Care* **2022**, *45*, 576–584, doi:10.2337/dc21-1970.
124. Kackley, M.L.; Short, J.A.; Hyde, P.N.; LaFountain, R.A.; Buga, A.; Miller, V.J.; Dickerson, R.M.; Sapper, T.N.; Barnhart, E.C.; Krishnan, D.; et al. A Pre-Workout Supplement of Ketone Salts, Caffeine, and Amino Acids Improves High-Intensity Exercise Performance in Keto-Naïve and Keto-Adapted Individuals. *J. Am. Coll. Nutr.* **2020**, *39*, 290–300, doi:10.1080/07315724.2020.1752846.
125. Kakoschke, N.; Zajac, I.T.; Tay, J.; Luscombe-Marsh, N.D.; Thompson, C.H.; Noakes, M.; Buckley, J.D.; Wittert, G.; Brinkworth, G.D. Effects of very low-carbohydrate vs. high-carbohydrate weight loss diets on psychological health in adults with obesity and type 2 diabetes: a 2-year randomized controlled trial. *Eur. J. Nutr.* **2021**, *60*, 4251–4262, doi:10.1007/s00394-021-02587-z.
126. Katsuya, S.; Kawata, Y.; Goto, T.; Tsubota, J. Daily Intake of D-β-Hydroxybutyric Acid (D-BHB) Reduces Body Fat in Japanese Adult Participants: A Randomized, Double-Blind, Placebo-Controlled Study. *J. Nutr. Sci. Vitaminol. (Tokyo)* **2023**, *69*, 121–128, doi:10.3177/jnsv.69.121.
127. Kenig, S.; Petelin, A.; Poklar Vatovec, T.; Mohorko, N.; Jenko-Pražnikar, Z. Assessment of micronutrients in a 12-wk ketogenic diet in obese adults. *Nutrition* **2019**, *67-68*, 110522, doi:10.1016/j.nut.2019.06.003.
128. Khodabakhshi, A.; Akbari, M.E.; Mirzaei, H.R.; Mehrad-Majd, H.; Kalamian, M.; Davoodi, S.H. Feasibility, Safety, and Beneficial Effects of MCT-Based Ketogenic Diet for Breast Cancer Treatment: A Randomized Controlled Trial Study. *Nutr. Cancer* **2020**, *72*, 627–634, doi:10.1080/01635581.2019.1650942.
129. Khodabakhshi, A.; Akbari, M.E.; Mirzaei, H.R.; Seyfried, T.N.; Kalamian, M.; Davoodi, S.H. Effects of Ketogenic metabolic therapy on patients with breast cancer: A randomized controlled clinical trial. *Clin. Nutr.* **2021**, *40*, 751–758, doi:10.1016/j.clnu.2020.06.028.
130. Khodabakhshi, A.; Seyfried, T.N.; Kalamian, M.; Beheshti, M.; Davoodi, S.H. Does a ketogenic diet have beneficial effects on quality of life, physical activity or biomarkers in patients with breast cancer: a randomized controlled clinical trial. *Nutr. J.* **2020**, *19*, 87, doi:10.1186/s12937-020-00596-y.
131. Kim, E.R.; Kim, S.R.; Cho, W.; Lee, S.-G.; Kim, S.H.; Kim, J.H.; Choi, E.; Kim, J.-H.; Yu, J.-W.; Lee, B.-W.; et al. Short Term Isocaloric Ketogenic Diet Modulates NLRP3 Inflammasome Via B-hydroxybutyrate and Fibroblast Growth Factor 21. *Front. Immunol.* **2022**, *13*, 843520, doi:10.3389/fimmu.2022.843520.
132. Kirkham, A.A.; King, K.; Joy, A.A.; Pelletier, A.B.; Mackey, J.R.; Young, K.; Zhu, X.; Meza-Junco, J.; Basi, S.K.; Hiller, J.P.; et al. Rationale and design of the Diet Restriction and Exercise-induced Adaptations in Metastatic breast cancer (DREAM) study: a 2-arm, parallel-group, phase II, randomized control trial of a short-term, calorie-restricted, and ketogenic diet plus exercise during intravenous chemotherapy versus usual care. *BMC Cancer* **2021**, *21*, 1093, doi:10.1186/s12885-021-08808-2.
133. Kishk, N.A.; Yousof, H.Z.; Ebraheim, A.M.; Elkholy, T.A.F.A.; Soliman, S.H.; Mohammed, R.A.; Shamloul, R.M. The effect of ketogenic diet escalation in adolescents and adults with drug-resistant epilepsy: a prospective study. *Nutr. Neurosci.* **2022**, *25*, 2023–2032, doi:10.1080/1028415X.2021.1927604.
134. Klejc, K.; Cruz-Almeida, Y.; Sheffler, J.L. Addressing Pain Using a Mediterranean Ketogenic Nutrition Program in Older Adults with Mild Cognitive Impairment. *J. Pain Res.* **2024**, *17*, 1867–1880, doi:10.2147/JPR.S451236.
135. Klement, R.J.; Champ, C.E.; Kämmerer, U.; Koebrunner, P.S.; Krage, K.; Schäfer, G.; Weigel, M.; Sweeney, R.A. Impact of a ketogenic diet intervention during radiotherapy on body composition: III-final results of the KETOCOMP study for breast cancer patients. *Breast Cancer Res.* **2020**, *22*, 94, doi:10.1186/s13058-020-01331-5.
136. Klement, R.J.; Koebrunner, P.S.; Meyer, D.; Kanzler, S.; Sweeney, R.A. Impact of a ketogenic diet intervention during radiotherapy on body composition: IV. Final results of the KETOCOMP study for rectal cancer patients. *Clin. Nutr.* **2021**, *40*, 4674–4684, doi:10.1016/j.clnu.2021.05.015.
137. Klement, R.J.; Meyer, D.; Kanzler, S.; Sweeney, R.A. Ketogenic diets consumed during radio-chemotherapy have beneficial effects on quality of life and metabolic health in patients with rectal cancer. *Eur. J. Nutr.* **2022**, *61*, 69–84, doi:10.1007/s00394-021-02615-y.
138. Klement, R.J.; Sweeney, R.A. Impact of a ketogenic diet intervention during radiotherapy on body composition: V. Final results of the KETOCOMP study for head and neck cancer patients. *Strahlenther. Onkol.* **2022**, *198*, 981–993, doi:10.1007/s00066-022-01941-2.

139. Klement, R.J.; Weigel, M.M.; Sweeney, R.A. A ketogenic diet consumed during radiotherapy improves several aspects of quality of life and metabolic health in women with breast cancer. *Clin. Nutr.* **2021**, *40*, 4267–4274, doi:10.1016/j.clnu.2021.01.023.
140. Koh, S.; Kim, T.-J.; Shin, H.-B.; Kim, H.K.; Park, B.; Moon, S.Y.; Kim, B.G.; Huh, K.; Choi, J.Y. Expanding Indications for a Ketogenic Diet as an Adjuvant Therapy in Adult Refractory Status Epilepticus: an Exploratory Study Using Moderation Analysis. *Neurotherapeutics* **2022**, *19*, 1526–1534, doi:10.1007/s13311-022-01282-z.
141. Kolivas, D.; Fraser, L.; Schweitzer, R.; Brukner, P.; Moschonis, G. Effectiveness of a Digitally Delivered Continuous Care Intervention (Defeat Diabetes) on Type 2 Diabetes Outcomes: A 12-Month Single-Arm, Pre-Post Intervention Study. *Nutrients* **2023**, *15*, doi:10.3390/nu15092153.
142. Krauss, R.M.; Fisher, L.M.; King, S.M.; Gardner, C.D. Changes in soluble LDL receptor and lipoprotein fractions in response to diet in the DIETFITS weight loss study. *J. Lipid Res.* **2024**, *65*, 100503, doi:10.1016/j.jlr.2024.100503.
143. Kverneland, M.; Nakken, K.O.; Hofoss, D.; Skogan, A.H.; Iversen, P.O.; Selmer, K.K.; Lossius, M.I. Health-related quality of life in adults with drug-resistant focal epilepsy treated with modified Atkins diet in a randomized clinical trial. *Epilepsia* **2023**, *64*, e69-e74, doi:10.1111/epi.17585.
144. Kysel, P.; Haluzíková, D.; Doležalová, R.P.; Laňková, I.; Lacinová, Z.; Kasperová, B.J.; Trnovská, J.; Hrádková, V.; Mráz, M.; Vilikus, Z.; et al. The Influence of Cyclical Ketogenic Reduction Diet vs. Nutritionally Balanced Reduction Diet on Body Composition, Strength, and Endurance Performance in Healthy Young Males: A Randomized Controlled Trial. *Nutrients* **2020**, *12*, doi:10.3390/nu12092832.
145. Kysel, P.; Haluzíková, D.; Pleyerová, I.; Řezníčková, K.; Laňková, I.; Lacinová, Z.; Havrlantová, T.; Mráz, M.; Kasperová, B.J.; Kovářová, V.; et al. Different Effects of Cyclical Ketogenic vs. Nutritionally Balanced Reduction Diet on Serum Concentrations of Myokines in Healthy Young Males Undergoing Combined Resistance/Aerobic Training. *Nutrients* **2023**, *15*, doi:10.3390/nu15071720.
146. Lakshminarayanan, K.; Agarawal, A.; Panda, P.K.; Sinha, R.; Tripathi, M.; Pandey, R.M.; Gulati, S. Efficacy of low glycemic index diet therapy (LGIT) in children aged 2-8 years with drug-resistant epilepsy: A randomized controlled trial. *Epilepsy Res.* **2021**, *171*, 106574, doi:10.1016/j.eplepsyres.2021.106574.
147. Lambadiari, V.; Katsimbri, P.; Kountouri, A.; Korakas, E.; Papathanasi, A.; Maratou, E.; Pavlidis, G.; Pliouta, L.; Ikonomidis, I.; Malisova, S.; et al. The Effect of a Ketogenic Diet versus Mediterranean Diet on Clinical and Biochemical Markers of Inflammation in Patients with Obesity and Psoriatic Arthritis: A Randomized Crossover Trial. *Int. J. Mol. Sci.* **2024**, *25*, doi:10.3390/ijms25052475.
148. Landry, M.J.; Crimarco, A.; Perelman, D.; Durand, L.R.; Petlura, C.; Aronica, L.; Robinson, J.L.; Kim, S.H.; Gardner, C.D. Adherence to Ketogenic and Mediterranean Study Diets in a Crossover Trial: The Keto-Med Randomized Trial. *Nutrients* **2021**, *13*, doi:10.3390/nu13030967.
149. Lauritsen, K.M.; Søndergaard, E.; Luong, T.V.; Møller, N.; Gormsen, L.C. Acute Hyperketonemia Does Not Affect Glucose or Palmitate Uptake in Abdominal Organs or Skeletal Muscle. *J. Clin. Endocrinol. Metab.* **2020**, *105*, doi:10.1210/clinem/dgaa122.
150. Lauritzen, E.S.; Svart, M.V.; Voss, T.; Møller, N.; Bjerre, M. Impact of Acutely Increased Endogenous- and Exogenous Ketone Bodies on FGF21 Levels in Humans. *Endocr. Res.* **2021**, *46*, 20–27, doi:10.1080/07435800.2020.1831015.
151. Lawford, B.; Hinman, R.S.; Jones, S.; Keating, C.; Brown, C.; Bennell, K.L. 'The fact that I know I can do it is quite a motivator now': a qualitative study exploring experiences maintaining weight loss 6 months after completing a weight loss programme for knee osteoarthritis. *BMJ Open* **2023**, *13*, e068157, doi:10.1136/bmjopen-2022-068157.
152. Lee, J.E.; Titcomb, T.J.; Bisht, B.; Rubenstein, L.M.; Louison, R.; Wahls, T.L. A Modified MCT-Based Ketogenic Diet Increases Plasma β-Hydroxybutyrate but Has Less Effect on Fatigue and Quality of Life in People with Multiple Sclerosis Compared to a Modified Paleolithic Diet: A Waitlist-Controlled, Randomized Pilot Study. *J. Am. Coll. Nutr.* **2021**, *40*, 13–25, doi:10.1080/07315724.2020.1734988.
153. Li, J.; Bai, W.-P.; Jiang, B.; Bai, L.-R.; Gu, B.; Yan, S.-X.; Li, F.-Y.; Huang, B. Ketogenic diet in women with polycystic ovary syndrome and liver dysfunction who are obese: A randomized, open-label, parallel-group, controlled pilot trial. *J. Obstet. Gynaecol. Res.* **2021**, *47*, 1145–1152, doi:10.1111/jog.14650.
154. Li, S.; Lin, G.; Chen, J.; Chen, Z.; Xu, F.; Zhu, F.; Zhang, J.; Yuan, S. The effect of periodic ketogenic diet on newly diagnosed overweight or obese patients with type 2 diabetes. *BMC Endocr. Disord.* **2022**, *22*, 34, doi:10.1186/s12902-022-00947-2.

155. Li, S.; Yuan, S.; Lin, G.; Zhang, J. Effects of a two meals-a-day ketogenic diet on newly diagnosed obese patients with type 2 diabetes mellitus: A retrospective observational study. *Medicine (Baltimore)* **2023**, *102*, e35753, doi:10.1097/MD.0000000000035753.
156. Li, X.; Shi, Z.; Byanyima, J.; Morgan, P.T.; van der Veen, J.-W.; Zhang, R.; Deneke, E.; Wang, G.-J.; Volkow, N.D.; Wiers, C.E. Brain glutamate and sleep efficiency associations following a ketogenic diet intervention in individuals with Alcohol Use Disorder. *Drug Alcohol Depend. Rep.* **2022**, *5*, doi:10.1016/j.dadr.2022.100092.
157. Lin, P.-H.; Howard, L.; Freedland, S.J. Impact of Low Carbohydrate Diet on Self-Report Fatigue and Weakness in Prostate Cancer Patients. *J. Urol.* **2021**, *206*, 499–501, doi:10.1097/JU.0000000000001780.
158. Lin, P.-H.; Howard, L.; Freedland, S.J. Weight loss via a low-carbohydrate diet improved the intestinal permeability marker, zonulin, in prostate cancer patients. *Ann. Med.* **2022**, *54*, 1221–1225, doi:10.1080/07853890.2022.2069853.
159. Lindqvist, C.; Holmer, M.; Hagström, H.; Petersson, S.; Tillander, V.; Brismar, T.B.; Stål, P. Macronutrient composition and its effect on body composition changes during weight loss therapy in patients with non-alcoholic fatty liver disease: Secondary analysis of a randomized controlled trial. *Nutrition* **2023**, *110*, 111982, doi:10.1016/j.nut.2023.111982.
160. Liu, F.; Peng, J.; Zhu, C.; Xiao, H.; He, F.; Yin, F.; Chen, C. Efficacy of the ketogenic diet in Chinese children with Dravet syndrome: A focus on neuropsychological development. *Epilepsy Behav.* **2019**, *92*, 98–102, doi:10.1016/j.yebeh.2018.12.016.
161. Løkken, N.; Hansen, K.K.; Storgaard, J.H.; Ørngreen, M.C.; Quinlivan, R.; Vissing, J. Titrating a modified ketogenic diet for patients with McArdle disease: A pilot study. *J. Inherit. Metab. Dis.* **2020**, *43*, 778–786, doi:10.1002/jimd.12223.
162. Løkken, N.; Nielsen, M.R.; Stemmerik, M.G.; Ellerton, C.; Revsbech, K.L.; Macrae, M.; Slipsager, A.; Krett, B.; Beha, G.H.; Emanuelsson, F.; et al. Can a modified ketogenic diet be a nutritional strategy for patients with McArdle disease? Results from a randomized, single-blind, placebo-controlled, cross-over study. *Clin. Nutr.* **2023**, *42*, 2124–2137, doi:10.1016/j.clnu.2023.09.006.
163. Løkken, N.; Storgaard, J.H.; Revsbech, K.L.; Voermans, N.C.; van Hall, G.; Vissing, J.; Ørngreen, M.C. No effect of oral ketone ester supplementation on exercise capacity in patients with McArdle disease and healthy controls: A randomized placebo-controlled cross-over study. *J. Inherit. Metab. Dis.* **2022**, *45*, 502–516, doi:10.1002/jimd.12484.
164. Lovati, C.; d'Alessandro, C.M.; Della Ventura, S.; Muzio, F.; Pantoni, L. Ketogenic diet in refractory migraine: possible efficacy and role of ketone bodies-a pilot experience. *Neurol. Sci.* **2022**, *43*, 6479–6485, doi:10.1007/s10072-022-06311-5.
165. Lowe, H.; Keller, A.E.; Tanzini, E.; Aimola, S.; Liu, Y.M.C.; Zak, M.; Chan, V.; Kobayashi, J.; Donner, E.J. Ketonuria and Seizure Control in the Medium Chain Triglyceride and Classic Ketogenic Diets. *Can. J. Neurol. Sci.* **2022**, *49*, 433–436, doi:10.1017/cjn.2021.122.
166. Lu, J.F.; Zhu, M.Q.; Xia, B.; Zhang, N.N.; Liu, X.P.; Liu, H.; Zhang, R.X.; Xiao, J.Y.; Yang, H.; Zhang, Y.Q.; et al. GDF15 is a major determinant of ketogenic diet-induced weight loss. *Cell Metab.* **2023**, *35*, 2165-2182.e7, doi:10.1016/j.cmet.2023.11.003.
167. Lundanes, J.; Sandnes, F.; Gjeilo, K.H.; Hansson, P.; Salater, S.; Martins, C.; Nymo, S. Effect of a low-carbohydrate diet on pain and quality of life in female patients with lipedema: a randomized controlled trial. *Obesity (Silver Spring)* **2024**, *32*, 1071–1082, doi:10.1002/oby.24026.
168. Lundsgaard, A.-M.; Fritzen, A.M.; Sjøberg, K.A.; Kleinert, M.; Richter, E.A.; Kiens, B. Small Amounts of Dietary Medium-Chain Fatty Acids Protect Against Insulin Resistance During Caloric Excess in Humans. *Diabetes* **2021**, *70*, 91–98, doi:10.2337/db20-0582.
169. Luong, T.V.; Pedersen, M.G.B.; Abild, C.B.; Cunnane, S.C.; Croteau, E.; Lauritsen, K.M.; Kjaerulff, M.L.G.; Tolbod, L.P.; Møller, N.; Søndergaard, E.; et al. A ketogenic diet lowers myocardial fatty acid oxidation but does not affect oxygen consumption: a study in overweight humans. *Obesity (Silver Spring)* **2024**, *32*, 506–516, doi:10.1002/oby.23967.
170. Lyman, K.S.; Athinarayanan, S.J.; McKenzie, A.L.; Pearson, C.L.; Adams, R.N.; Hallberg, S.J.; McCarter, J.P.; Volek, J.S.; Phinney, S.D.; Andrawis, J.P. Continuous care intervention with carbohydrate restriction improves physical function of the knees among patients with type 2 diabetes: a non-randomized study. *BMC Musculoskelet. Disord.* **2022**, *23*, 297, doi:10.1186/s12891-022-05258-0.

171. Lyngstad, A.; Nymo, S.; Coutinho, S.R.; Rehfeld, J.F.; Truby, H.; Kulseng, B.; Martins, C. Investigating the effect of sex and ketosis on weight-loss-induced changes in appetite. *Am. J. Clin. Nutr.* **2019**, *109*, 1511–1518, doi:10.1093/ajcn/nqz002.
172. Ma, D.C.; Anderson, C.M.; Rodman, S.N.; Buranasudja, V.; McCormick, M.L.; Davis, A.; Loth, E.; Bodeker, K.L.; Ahmann, L.; Parkhurst, J.R.; et al. Ketogenic Diet with Concurrent Chemoradiation in Head and Neck Squamous Cell Carcinoma: Preclinical and Phase 1 Trial Results. *Radiat. Res.* **2021**, *196*, 213–224, doi:10.1667/RADE-20-00150.1.
173. Manral, M.; Dwivedi, R.; Gulati, S.; Kaur, K.; Nehra, A.; Pandey, R.M.; Upadhyay, A.D.; Sapra, S.; Tripathi, M. Safety, Efficacy, and Tolerability of Modified Atkins Diet in Persons With Drug-Resistant Epilepsy: A Randomized Controlled Trial. *Neurology* **2023**, *100*, e1376-e1385, doi:10.1212/WNL.0000000000206776.
174. Marchi, F. de; Collo, A.; Scognamiglio, A.; Cavaletto, M.; Bozzi Cionci, N.; Biroli, G.; Di Gioia, D.; Riso, S.; Mazzini, L. Study protocol on the safety and feasibility of a normocaloric ketogenic diet in people with amyotrophic lateral sclerosis. *Nutrition* **2022**, *94*, 111525, doi:10.1016/j.nut.2021.111525.
175. Marchiò, M.; Roli, L.; Giordano, C.; Trenti, T.; Guerra, A.; Biagini, G. Decreased ghrelin and des-acyl ghrelin plasma levels in patients affected by pharmacoresistant epilepsy and maintained on the ketogenic diet. *Clin. Nutr.* **2019**, *38*, 954–957, doi:10.1016/j.clnu.2018.03.009.
176. Martin-McGill, K.J.; Marson, A.G.; Tudur Smith, C.; Young, B.; Mills, S.J.; Cherry, M.G.; Jenkinson, M.D. Ketogenic diets as an adjuvant therapy for glioblastoma (KEATING): a randomized, mixed methods, feasibility study. *J. Neurooncol.* **2020**, *147*, 213–227, doi:10.1007/s11060-020-03417-8.
177. Martins, C.; Nymo, S.; Aukan, M.I.; Roekenes, J.A.; Coutinho, S.R.; Hunter, G.R.; Gower, B.A. Association between ß-Hydroxybutyrate Plasma Concentrations after Hypocaloric Ketogenic Diets and Changes in Body Composition. *J. Nutr.* **2023**, *153*, 1944–1949, doi:10.1016/j.tjnut.2023.05.010.
178. Maunder, E.; Dulson, D.K.; Shaw, D.M. Autonomic and Perceptual Responses to Induction of a Ketogenic Diet in Free-Living Endurance Athletes: A Randomized, Crossover Trial. *Int. J. Sports Physiol. Perform.* **2021**, *16*, 1603–1609, doi:10.1123/ijspp.2020-0814.
179. McCullough, D.; Harrison, T.; Enright, K.J.; Amirabdollahian, F.; Mazidi, M.; Lane, K.E.; Stewart, C.E.; Davies, I.G. The Effect of Carbohydrate Restriction on Lipids, Lipoproteins, and Nuclear Magnetic Resonance-Based Metabolites: CALIBER, a Randomised Parallel Trial. *Nutrients* **2023**, *15*, doi:10.3390/nu15133002.
180. McCullough, D.; Harrison, T.; Boddy, L.M.; Enright, K.J.; Amirabdollahian, F.; Schmidt, M.A.; Doenges, K.; Quinn, K.; Reisdorph, N.; Mazidi, M.; et al. The Effect of Dietary Carbohydrate and Fat Manipulation on the Metabolome and Markers of Glucose and Insulin Metabolism: A Randomised Parallel Trial. *Nutrients* **2022**, *14*, doi:10.3390/nu14183691.
181. McKenzie, A.L.; Athinarayanan, S.J.; McCue, J.J.; Adams, R.N.; Keyes, M.; McCarter, J.P.; Volek, J.S.; Phinney, S.D.; Hallberg, S.J. Type 2 Diabetes Prevention Focused on Normalization of Glycemia: A Two-Year Pilot Study. *Nutrients* **2021**, *13*, doi:10.3390/nu13030749.
182. McNelly, A.; Langan, A.; Bear, D.E.; Page, A.; Martin, T.; Seidu, F.; Santos, F.; Rooney, K.; Liang, K.; Heales, S.J.; et al. A pilot study of alternative substrates in the critically Ill subject using a ketogenic feed. *Nat. Commun.* **2023**, *14*, 8345, doi:10.1038/s41467-023-42659-8.
183. Michalczyk, M.M.; Chycki, J.; Zajac, A.; Maszczyk, A.; Zydek, G.; Langfort, J. Anaerobic Performance after a Low-Carbohydrate Diet (LCD) Followed by 7 Days of Carbohydrate Loading in Male Basketball Players. *Nutrients* **2019**, *11*, doi:10.3390/nu11040778.
184. Michalczyk, M.M.; Klonek, G.; Maszczyk, A.; Zajac, A. The Effects of a Low Calorie Ketogenic Diet on Glycaemic Control Variables in Hyperinsulinemic Overweight/Obese Females. *Nutrients* **2020**, *12*, doi:10.3390/nu12061854.
185. Moasses-Ghafari, B.; Fallahi, B.; Esfehani, A.F.; Eftekhari, M.; Rahmani, K.; Eftekhari, A.; Geramifar, P. Effect of Diet on Physiologic Bowel 18F-FDG Uptake. *J. Nucl. Med. Technol.* **2021**, *49*, 241–245, doi:10.2967/jnmt.120.257857.
186. Moriconi, E.; Camajani, E.; Fabbri, A.; Lenzi, A.; Caprio, M. Very-Low-Calorie Ketogenic Diet as a Safe and Valuable Tool for Long-Term Glycemic Management in Patients with Obesity and Type 2 Diabetes. *Nutrients* **2021**, *13*, doi:10.3390/nu13030758.
187. Morrison, S.A.; Fazeli, P.L.; Gower, B.; Willig, A.L.; Younger, J.; Sneed, N.M.; Vance, D.E. Cognitive Effects of a Ketogenic Diet on Neurocognitive Impairment in Adults Aging With HIV: A Pilot Study. *J. Assoc. Nurses AIDS Care* **2020**, *31*, 312–324, doi:10.1097/JNC.0000000000000110.

188. Mose, M.; Brodersen, K.; Rittig, N.; Schmidt, J.; Jessen, N.; Mikkelsen, U.R.; Jørgensen, J.O.L.; Møller, N. Anabolic effects of oral leucine-rich protein with and without β-hydroxybutyrate on muscle protein metabolism in a novel clinical model of systemic inflammation-a randomized crossover trial. *Am. J. Clin. Nutr.* **2021**, *114*, 1159–1172, doi:10.1093/ajcn/nqab148.
189. Mu, C.; Corley, M.J.; Lee, R.W.Y.; Wong, M.; Pang, A.; Arakaki, G.; Miyamoto, R.; Rho, J.M.; Mickiewicz, B.; Dowlatabadi, R.; et al. Metabolic Framework for the Improvement of Autism Spectrum Disorders by a Modified Ketogenic Diet: A Pilot Study. *J. Proteome Res.* **2020**, *19*, 382–390, doi:10.1021/acs.jproteome.9b00581.
190. Murtaza, N.; Burke, L.M.; Vlahovich, N.; Charlesson, B.; O' Neill, H.; Ross, M.L.; Campbell, K.L.; Krause, L.; Morrison, M. The Effects of Dietary Pattern during Intensified Training on Stool Microbiota of Elite Race Walkers. *Nutrients* **2019**, *11*, doi:10.3390/nu11020261.
191. Myette-Côté, É.; Caldwell, H.G.; Ainslie, P.N.; Clarke, K.; Little, J.P. A ketone monoester drink reduces the glycemic response to an oral glucose challenge in individuals with obesity: a randomized trial. *Am. J. Clin. Nutr.* **2019**, *110*, 1491–1501, doi:10.1093/ajcn/nqz232.
192. Myette-Côté, É.; St-Pierre, V.; Beaulieu, S.; Castellano, C.-A.; Fortier, M.; Plourde, M.; Bocti, C.; Fulop, T.; Cunnane, S.C. The effect of a 6-month ketogenic medium-chain triglyceride supplement on plasma cardiometabolic and inflammatory markers in mild cognitive impairment. *Prostaglandins Leukot. Essent. Fatty Acids* **2021**, *169*, 102236, doi:10.1016/j.plefa.2020.102236.
193. Najafabadi, M.S.; Moludi, J.; Salimi, Y.; Saber, A. A comparison of the portfolio low-carbohydrate diet and the ketogenic diet in overweight and obese women with polycystic ovary syndrome: study protocol for a randomized controlled trial. *Trials* **2023**, *24*, 509, doi:10.1186/s13063-023-07569-6.
194. Nakagata, T.; Tamura, Y.; Kaga, H.; Sato, M.; Yamasaki, N.; Someya, Y.; Kadowaki, S.; Sugimoto, D.; Satoh, H.; Kawamori, R.; et al. Ingestion of an exogenous ketone monoester improves the glycemic response during oral glucose tolerance test in individuals with impaired glucose tolerance: A cross-over randomized trial. *J. Diabetes Investig.* **2021**, *12*, 756–762, doi:10.1111/jdi.13423.
195. Nakamura, K.; Hagihara, K.; Nagai, N.; Egashira, R.; Takeuchi, M.; Nakano, M.; Saito, H.; Moriguchi, M.; Tonari, S.; Watanabe, S.; et al. Ketogenic Effects of Multiple Doses of a Medium Chain Triglycerides Enriched Ketogenic Formula in Healthy Men under the Ketogenic Diet: A Randomized, Double-Blinded, Placebo-Controlled Study. *Nutrients* **2022**, *14*, doi:10.3390/nu14061199.
196. Nuttall, F.Q.; Almokayyad, R.M.; Gannon, M.C. Circulating lipids in men with type 2 diabetes following 3 days on a carbohydrate-free diet versus 3 days of fasting. *Physiol. Rep.* **2020**, *8*, e14569, doi:10.14814/phy2.14569.
197. Nybacka, S.; Törnblom, H.; Josefsson, A.; Hreinsson, J.P.; Böhn, L.; Frändemark, Å.; Weznaver, C.; Störsrud, S.; Simrén, M. A low FODMAP diet plus traditional dietary advice versus a low-carbohydrate diet versus pharmacological treatment in irritable bowel syndrome (CARIBS): a single-centre, single-blind, randomised controlled trial. *Lancet Gastroenterol. Hepatol.* **2024**, *9*, 507–520, doi:10.1016/S2468-1253(24)00045-1.
198. Oehm, S.; Steinke, K.; Schmidt, J.; Arjune, S.; Todorova, P.; Heinrich Lindemann, C.; Wöstmann, F.; Meyer, F.; Siedek, F.; Weimbs, T.; et al. RESET-PKD: a pilot trial on short-term ketogenic interventions in autosomal dominant polycystic kidney disease. *Nephrol. Dial. Transplant* **2023**, *38*, 1623–1635, doi:10.1093/ndt/gfac311.
199. Ofir, N.; Mizrakli, Y.; Greenshpan, Y.; Gepner, Y.; Sharabi, O.; Tsaban, G.; Zelicha, H.; Yaskolka Meir, A.; Ceglarek, U.; Stumvoll, M.; et al. Vertebrae but not femur marrow fat transiently decreases in response to body weight loss in an 18-month randomized control trial. *Bone* **2023**, *171*, 116727, doi:10.1016/j.bone.2023.116727.
200. O'Neill, B.V.; Dodds, C.M.; Miller, S.R.; Gupta, A.; Lawrence, P.; Bullman, J.; Chen, C.; Dewit, O.; Kumar, S.; Dustagheer, M.; et al. The effects of GSK2981710, a medium-chain triglyceride, on cognitive function in healthy older participants: A randomised, placebo-controlled study. *Hum. Psychopharmacol.* **2019**, *34*, e2694, doi:10.1002/hup.2694.
201. Ota, M.; Matsuo, J.; Ishida, I.; Takano, H.; Yokoi, Y.; Hori, H.; Yoshida, S.; Ashida, K.; Nakamura, K.; Takahashi, T.; et al. Effects of a medium-chain triglyceride-based ketogenic formula on cognitive function in patients with mild-to-moderate Alzheimer's disease. *Neurosci. Lett.* **2019**, *690*, 232–236, doi:10.1016/j.neulet.2018.10.048.
202. Ozoran, H.; Matheou, M.; Dyson, P.; Karpe, F.; Tan, G.D. Type 1 diabetes and low carbohydrate diets-Defining the degree of nutritional ketosis. *Diabet. Med.* **2023**, *40*, e15178, doi:10.1111/dme.15178.

203. Panda, P.K.; Chakrabarty, B.; Jauhari, P.; Sharawat, I.K.; Agarwal, A.; Jain, V.; Pandey, R.M.; Gulati, S. Efficacy of daily versus intermittent low glycemic index therapy diet in children with drug-resistant epilepsy: A randomized controlled trial. *Epilepsy Res.* **2024**, *201*, 107322, doi:10.1016/j.eplepsyres.2024.107322.
204. Paoli, A.; Cenci, L.; Pompei, P.; Sahin, N.; Bianco, A.; Neri, M.; Caprio, M.; Moro, T. Effects of Two Months of Very Low Carbohydrate Ketogenic Diet on Body Composition, Muscle Strength, Muscle Area, and Blood Parameters in Competitive Natural Body Builders. *Nutrients* **2021**, *13*, doi:10.3390/nu13020374.
205. Paoli, A.; Mancin, L.; Giacona, M.C.; Bianco, A.; Caprio, M. Effects of a ketogenic diet in overweight women with polycystic ovary syndrome. *J. Transl. Med.* **2020**, *18*, 104, doi:10.1186/s12967-020-02277-0.
206. Pauley, M.; Mays, C.; Bailes, J.R.; Schwartzman, M.L.; Castle, M.; McCoy, M.; Patick, C.; Preston, D.; Nudelman, M.J.R.; Denning, K.L.; et al. Carbohydrate-Restricted Diet: A Successful Strategy for Short-Term Management in Youth with Severe Obesity-An Observational Study. *Metab. Syndr. Relat. Disord.* **2021**, *19*, 281–287, doi:10.1089/met.2020.0078.
207. Perissiou, M.; Borkoles, E.; Kobayashi, K.; Polman, R. The Effect of an 8 Week Prescribed Exercise and Low-Carbohydrate Diet on Cardiorespiratory Fitness, Body Composition and Cardiometabolic Risk Factors in Obese Individuals: A Randomised Controlled Trial. *Nutrients* **2020**, *12*, doi:10.3390/nu12020482.
208. Petrisko, M.; Kloss, R.; Bradley, P.; Birrenkott, E.; Spindler, A.; Clayton, Z.S.; Kern, M. Biochemical, Anthropometric, and Physiological Responses to Carbohydrate-Restricted Diets Versus a Low-Fat Diet in Obese Adults: A Randomized Crossover Trial. *J. Med. Food* **2020**, *23*, 206–214, doi:10.1089/jmf.2019.0266.
209. Phillips, M.C.L.; Deprez, L.M.; Mortimer, G.M.N.; Murtagh, D.K.J.; McCoy, S.; Mylchreest, R.; Gilbertson, L.J.; Clark, K.M.; Simpson, P.V.; McManus, E.J.; et al. Randomized crossover trial of a modified ketogenic diet in Alzheimer's disease. *Alzheimers. Res. Ther.* **2021**, *13*, 51, doi:10.1186/s13195-021-00783-x.
210. Polito, R.; Valenzano, A.; Monda, V.; Cibelli, G.; Monda, M.; Messina, G.; Villano, I.; Messina, A. Heart Rate Variability and Sympathetic Activity Is Modulated by Very Low-Calorie Ketogenic Diet. *Int. J. Environ. Res. Public Health* **2022**, *19*, doi:10.3390/ijerph19042253.
211. Poorshiri, B.; Barzegar, M.; Tahmasebi, S.; Shiva, S.; Raeisi, S.; Ebadi, Z. The efficacy comparison of classic ketogenic diet and modified Atkins diet in children with refractory epilepsy: a clinical trial. *Acta Neurol. Belg.* **2021**, *121*, 483–487, doi:10.1007/s13760-019-01225-0.
212. Porper, K.; Shpatz, Y.; Plotkin, L.; Pechthold, R.G.; Talianski, A.; Champ, C.E.; Furman, O.; Shimoni-Sebag, A.; Symon, Z.; Amit, U.; et al. A Phase I clinical trial of dose-escalated metabolic therapy combined with concomitant radiation therapy in high-grade glioma. *J. Neurooncol.* **2021**, *153*, 487–496, doi:10.1007/s11060-021-03786-8.
213. Prado, E.; Magalhães-Neto, A.; Neto, J.R.; Bassini, A.; Cameron, L.-C. Caffeine decreases ammonemia in athletes using a ketogenic diet during prolonged exercise. *Nutrition* **2021**, *91-92*, 111377, doi:10.1016/j.nut.2021.111377.
214. Prins, P.J.; Noakes, T.D.; Welton, G.L.; Haley, S.J.; Esbenshade, N.J.; Atwell, A.D.; Scott, K.E.; Abraham, J.; Raabe, A.S.; Buxton, J.D.; et al. High Rates of Fat Oxidation Induced by a Low-Carbohydrate, High-Fat Diet, Do Not Impair 5-km Running Performance in Competitive Recreational Athletes. *J. Sports Sci. Med.* **2019**, *18*, 738–750.
215. Putananickal, N.; Gross, E.C.; Orsini, A.-L.; Schmidt, S.; Hafner, P.; Gocheva, V.; Nagy, S.; Henzi, B.C.; Rubino, D.; Vogt, D.R.; et al. Efficacy and safety of exogenous beta-hydroxybutyrate for preventive treatment in episodic migraine: A single-centred, randomised, placebo-controlled, double-blind crossover trial. *Cephalalgia* **2022**, *42*, 302–311, doi:10.1177/03331024211043792.
216. Rahmel, T.; Effinger, D.; Bracht, T.; Griep, L.; Koos, B.; Sitek, B.; Hübner, M.; Hirschberger, S.; Basten, J.; Timmesfeld, N.; et al. An open-label, randomized controlled trial to assess a ketogenic diet in critically ill patients with sepsis. *Sci. Transl. Med.* **2024**, *16*, eadn9285, doi:10.1126/scitranslmed.adn9285.
217. Rahmel, T.; Hübner, M.; Koos, B.; Wolf, A.; Willemsen, K.-M.; Strauß, G.; Effinger, D.; Adamzik, M.; Kreth, S. Impact of carbohydrate-reduced nutrition in septic patients on ICU: study protocol for a prospective randomised controlled trial. *BMJ Open* **2020**, *10*, e038532, doi:10.1136/bmjopen-2020-038532.
218. Rasmussen, E.; Patel, V.; Tideman, S.; Frech, R.; Frigerio, R.; Narayanan, J. Efficacy of supplemental MCT oil on seizure reduction of adult drug-resistant epilepsy - a single-center open-label pilot study. *Nutr. Neurosci.* **2023**, *26*, 535–539, doi:10.1080/1028415X.2022.2065816.
219. Ray, S.; Nathan, J.; Godhia, M. Efficacy and tolerability of classical and polyunsaturated fatty acids ketogenic diet in controlling paediatric refractory epilepsy - A randomized study. *Epilepsy Res.* **2024**, *204*, 107395, doi:10.1016/j.eplepsyres.2024.107395.

220. Riantarini, I.; Kim, H.D.; Ko, A.; Kim, S.H.; Kang, H.-C.; Lee, J.S.; Da Jung, E. Short- and long-term seizure-free outcomes of dietary treatment in infants according to etiology. *Seizure* **2019**, *71*, 100–104, doi:10.1016/j.seizure.2019.06.002.
221. Robinson, H.; Barrett, H.; Gomez-Arango, L.; McIntyre, H.D.; Callaway, L.; Dekker Nitert, M. Ketonuria Is Associated with Changes to the Abundance of Roseburia in the Gut Microbiota of Overweight and Obese Women at 16 Weeks Gestation: A Cross-Sectional Observational Study. *Nutrients* **2019**, *11*, doi:10.3390/nu11081836.
222. Romão Luz, I.; Pereira, C.; Garcia, P.; Ferreira, F.; Faria, A.; Macedo, C.; Diogo, L.; Robalo, C. Ketogenic Diet for Refractory Childhood Epilepsy: Beyond Seizures Control, the Experience of a Portuguese Pediatric Centre. *Acta Med. Port.* **2019**, *32*, 760–766, doi:10.20344/amp.12184.
223. Roy, M.; Edde, M.; Fortier, M.; Croteau, E.; Castellano, C.-A.; St-Pierre, V.; Vandenberghe, C.; Rheault, F.; Dadar, M.; Duchesne, S.; et al. A ketogenic intervention improves dorsal attention network functional and structural connectivity in mild cognitive impairment. *Neurobiol. Aging* **2022**, *115*, 77–87, doi:10.1016/j.neurobiolaging.2022.04.005.
224. Ruiz Herrero, J.; Cañedo Villarroya, E.; García Peñas, J.J.; García Alcolea, B.; Gómez Fernández, B.; Puerta Macfarland, L.A.; Pedrón Giner, C. Safety and Effectiveness of the Prolonged Treatment of Children with a Ketogenic Diet. *Nutrients* **2020**, *12*, doi:10.3390/nu12020306.
225. Ruiz Herrero, J.; Cañedo Villarroya, E.; García Peñas, J.J.; García Alcolea, B.; Gómez Fernández, B.; Puerta Macfarland, L.A.; Pedrón-Giner, C. Ketogenic dietary therapies for epilepsy: Experience in 160 patients over 18 years. *An. Pediatr. (Engl Ed)* **2022**, *96*, 511–522, doi:10.1016/j.anpede.2022.05.001.
226. Ruiz-Herrero, J.; Cañedo-Villarroya, E.; Pérez-Sebastián, I.; Bernardino-Cuesta, B.; Pedrón-Giner, C. Efficacy and safety of ketogenic dietary theraphies in infancy. A single-center experience in 42 infants less than two years of age. *Seizure* **2021**, *92*, 106–111, doi:10.1016/j.seizure.2021.08.018.
227. Sánchez, E.; Santos, M.-D.; Nuñez-Garcia, M.; Bueno, M.; Sajoux, I.; Yeramian, A.; Lecube, A. Randomized Clinical Trial to Evaluate the Morphological Changes in the Adventitial Vasa Vasorum Density and Biological Markers of Endothelial Dysfunction in Subjects with Moderate Obesity Undergoing a Very Low-Calorie Ketogenic Diet. *Nutrients* **2021**, *14*, doi:10.3390/nu14010033.
228. Saslow, L.R.; Eslamian, A.; Moran, P.; Hartogensis, W.; Mason, A.E.; Kim, S.; Bauer, D.C.; Griauzde, D.H.; Goldman, V.; Liu, V.; et al. Protocol for a randomized controlled trial comparing a very low-carbohydrate diet or moderate-carbohydrate plate-method diet for type 2 diabetes: the LEGEND (Lifestyle Education about Nutrition for Diabetes) trial. *Trials* **2023**, *24*, 463, doi:10.1186/s13063-023-07512-9.
229. Saslow, L.R.; Jones, L.M.; Sen, A.; Wolfson, J.A.; Diez, H.L.; O'Brien, A.; Leung, C.W.; Bayandorian, H.; Daubenmier, J.; Missel, A.L.; et al. Comparing Very Low-Carbohydrate vs DASH Diets for Overweight or Obese Adults With Hypertension and Prediabetes or Type 2 Diabetes: A Randomized Trial. *Ann. Fam. Med.* **2023**, *21*, 256–263, doi:10.1370/afm.2968.
230. Schiavo, L.; Pierro, R.; Asteria, C.; Calabrese, P.; Di Biasio, A.; Coluzzi, I.; Severino, L.; Giovanelli, A.; Pilone, V.; Silecchia, G. Low-Calorie Ketogenic Diet with Continuous Positive Airway Pressure to Alleviate Severe Obstructive Sleep Apnea Syndrome in Patients with Obesity Scheduled for Bariatric/Metabolic Surgery: a Pilot, Prospective, Randomized Multicenter Comparative Study. *Obes. Surg.* **2022**, *32*, 634–642, doi:10.1007/s11695-021-05811-1.
231. Schiavo, L.; Stefano, G. de; Persico, F.; Gargiulo, S.; Di Spirito, F.; Griguolo, G.; Petrucciani, N.; Fontas, E.; Iannelli, A.; Pilone, V. A Randomized, Controlled Trial Comparing the Impact of a Low-Calorie Ketogenic vs a Standard Low-Calorie Diet on Fat-Free Mass in Patients Receiving an Elipse™ Intragastric Balloon Treatment. *Obes. Surg.* **2021**, *31*, 1514–1523, doi:10.1007/s11695-020-05133-8.
232. Schoeler, N.E.; Marston, L.; Lyons, L.; Halsall, S.; Jain, R.; Titre-Johnson, S.; Balogun, M.; Heales, S.J.R.; Eaton, S.; Orford, M.; et al. Classic ketogenic diet versus further antiseizure medicine in infants with drug-resistant epilepsy (KIWE): a UK, multicentre, open-label, randomised clinical trial. *Lancet Neurol.* **2023**, *22*, 1113–1124, doi:10.1016/S1474-4422(23)00370-8.
233. Schreck, K.C.; Hsu, F.-C.; Berrington, A.; Henry-Barron, B.; Vizthum, D.; Blair, L.; Kossoff, E.H.; Easter, L.; Whitlow, C.T.; Barker, P.B.; et al. Feasibility and Biological Activity of a Ketogenic/Intermittent-Fasting Diet in Patients With Glioma. *Neurology* **2021**, *97*, e953-e963, doi:10.1212/WNL.0000000000012386.
234. Scott, B.E.; Laursen, P.B.; James, L.J.; Boxer, B.; Chandler, Z.; Lam, E.; Gascoyne, T.; Messenger, J.; Mears, S.A. The effect of 1,3-butanediol and carbohydrate supplementation on running performance. *J. Sci. Med. Sport* **2019**, *22*, 702–706, doi:10.1016/j.jsams.2018.11.027.

235. Scragg, J.; Morris, E.; Wane, S.; Noreik, M.; Jerome, D.; Yu, L.-M.; Galal, U.; Dyson, P.; Tan, G.D.; Fox, R.; et al. Dietary Approaches to the Management Of type 2 Diabetes (DIAMOND) in primary care: A protocol for a cluster randomised trial. *Contemp. Clin. Trials* **2023**, *129*, 107199, doi:10.1016/j.cct.2023.107199.
236. Sebastian, S.; Paul, A.; Joby, J.; Saijan, S.; Vilapurathu, J.K. Effect of high-dose intravenous ascorbic acid on cancer patients following ketogenic diet. *J. Cancer Res. Ther.* **2021**, *17*, 1583–1586, doi:10.4103/jcrt.JCRT_973_19.
237. Selvaraj, S.; Margulies, K.B.; Dugyala, S.; Schubert, E.; Tierney, A.; Arany, Z.; Pryma, D.A.; Shah, S.H.; Rame, J.E.; Kelly, D.P.; et al. Comparison of Exogenous Ketone Administration Versus Dietary Carbohydrate Restriction on Myocardial Glucose Suppression: A Crossover Clinical Trial. *J. Nucl. Med.* **2022**, *63*, 770–776, doi:10.2967/jnumed.121.262734.
238. Sethi, S.; Wakeham, D.; Ketter, T.; Hooshmand, F.; Bjornstad, J.; Richards, B.; Westman, E.; Krauss, R.M.; Saslow, L. Ketogenic Diet Intervention on Metabolic and Psychiatric Health in Bipolar and Schizophrenia: A Pilot Trial. *Psychiatry Res.* **2024**, *335*, 115866, doi:10.1016/j.psychres.2024.115866.
239. Shah, L.M.; Turner, Z.; Bessone, S.K.; Winesett, S.P.; Stanfield, A.; Kossoff, E.H. How often is antiseizure drug-free ketogenic diet therapy achieved? *Epilepsy Behav.* **2019**, *93*, 29–31, doi:10.1016/j.yebeh.2019.01.042.
240. Sharma, S.; Dabla, S.; Kaushik, J.S. Modified Atkins Diet vs. Ketogenic Diet in the Management of Children with Epileptic Spasms Refractory to First Line Treatment: An Open Labelled, Randomized Controlled Trial. *Indian J. Pediatr.* **2023**, *90*, 969–973, doi:10.1007/s12098-023-04527-7.
241. Sharma, S.; Goel, S.; Kapoor, D.; Garg, D.; Panda, I.; Elwadhi, A.; Patra, B.; Mukherjee, S.B.; Pemde, H. Evaluation of the Modified Atkins Diet for the Treatment of Epileptic Spasms Refractory to Hormonal Therapy: A Randomized Controlled Trial. *J. Child Neurol.* **2021**, *36*, 686–691, doi:10.1177/08830738211004747.
242. Shaw, D.M.; Henderson, L.; van den Berg, M. Cognitive, Sleep, and Autonomic Responses to Induction of a Ketogenic Diet in Military Personnel: A Pilot Study. *Aerosp. Med. Hum. Perform.* **2022**, *93*, 507–516, doi:10.3357/AMHP.6015.2022.
243. Shaw, D.M.; Keaney, L.; Maunder, E.; Dulson, D.K. Natural killer cell subset count and antigen-stimulated activation in response to exhaustive running following adaptation to a ketogenic diet. *Exp. Physiol.* **2023**, *108*, 706–714, doi:10.1113/EP090729.
244. Shaw, D.M.; Merien, F.; Braakhuis, A.; Keaney, L.; Dulson, D.K. Adaptation to a ketogenic diet modulates adaptive and mucosal immune markers in trained male endurance athletes. *Scand. J. Med. Sci. Sports* **2021**, *31*, 140–152, doi:10.1111/sms.13833.
245. Sheffler, J.L.; Kiosses, D.N.; He, Z.; Arjmandi, B.H.; Akhavan, N.S.; Klejc, K.; Naar, S. Improving Adherence to a Mediterranean Ketogenic Nutrition Program for High-Risk Older Adults: A Pilot Randomized Trial. *Nutrients* **2023**, *15*, doi:10.3390/nu15102329.
246. Siegmann, M.J.; Athinarayanan, S.J.; Hallberg, S.J.; McKenzie, A.L.; Bhanpuri, N.H.; Campbell, W.W.; McCarter, J.P.; Phinney, S.D.; Volek, J.S.; van Dort, C.J. Improvement in patient-reported sleep in type 2 diabetes and prediabetes participants receiving a continuous care intervention with nutritional ketosis. *Sleep Med.* **2019**, *55*, 92–99, doi:10.1016/j.sleep.2018.12.014.
247. Sjödin, A.; Hellström, F.; Sehlstedt, E.; Svensson, M.; Burén, J. Effects of a Ketogenic Diet on Muscle Fatigue in Healthy, Young, Normal-Weight Women: A Randomized Controlled Feeding Trial. *Nutrients* **2020**, *12*, doi:10.3390/nu12040955.
248. Sommersten, C.H.; Gjerde, E.S.; Laupsa-Borge, J.; Andersen, A.I.; Lawrence-Archer, L.; McCann, A.; Hansson, P.; Raza, G.S.; Herzig, K.H.; Lied, G.A.; et al. Relationship between Ketones, Ghrelin, and, Appetite on Isocaloric Diets with Varying Carbohydrate Quality and Amount: Results from a Randomized Controlled Trial in People with Obesity (CARBFUNC). *J. Nutr.* **2023**, *153*, 459–469, doi:10.1016/j.tjnut.2022.12.030.
249. Soto-Mota, A.; Norwitz, N.G.; Evans, R.; Clarke, K.; Barber, T.M. Exogenous ketosis in patients with type 2 diabetes: Safety, tolerability and effect on glycaemic control. *Endocrinol. Diabetes Metab.* **2021**, *4*, e00264, doi:10.1002/edm2.264.
250. Soto-Mota, A.; Vansant, H.; Evans, R.D.; Clarke, K. Safety and tolerability of sustained exogenous ketosis using ketone monoester drinks for 28 days in healthy adults. *Regul. Toxicol. Pharmacol.* **2019**, *109*, 104506, doi:10.1016/j.yrtph.2019.104506.
251. Souza Neves, G. de; Dos Santos Lunardi, M.; Papini Gabiatti, M.; Kurrle Rieger Venske, D.; Ribeiro, L.C.; Lin, K.; Dubois Moreira, J. Cardiometabolic risk and effectiveness of the modified Atkins Ketogenic Diet for

adult patients with pharmacoresistant epilepsies in a middle-income country. *Epilepsy Res.* **2020**, *160*, 106280, doi:10.1016/j.eplepsyres.2020.106280.
252. Stefan, M.; Sharp, M.; Gheith, R.; Lowery, R.; Wilson, J. The Effect of Exogenous Beta-Hydroxybutyrate Salt Supplementation on Metrics of Safety and Health in Adolescents. *Nutrients* **2021**, *13*, doi:10.3390/nu13030854.
253. Strath, L.J.; Jones, C.D.; Philip George, A.; Lukens, S.L.; Morrison, S.A.; Soleymani, T.; Locher, J.L.; Gower, B.A.; Sorge, R.E. The Effect of Low-Carbohydrate and Low-Fat Diets on Pain in Individuals with Knee Osteoarthritis. *Pain Med.* **2020**, *21*, 150–160, doi:10.1093/pm/pnz022.
254. Struik, N.A.; Brinkworth, G.D.; Thompson, C.H.; Buckley, J.D.; Wittert, G.; Luscombe-Marsh, N.D. Very Low and Higher Carbohydrate Diets Promote Differential Appetite Responses in Adults with Type 2 Diabetes: A Randomized Trial. *J. Nutr.* **2020**, *150*, 800–805, doi:10.1093/jn/nxz344.
255. Su, T.-Y.; Hung, P.-L.; Chen, C.; Lin, Y.-J.; Peng, S.-J. Graph Theory-Based Electroencephalographic Connectivity and Its Association with Ketogenic Diet Effectiveness in Epileptic Children. *Nutrients* **2021**, *13*, doi:10.3390/nu13072186.
256. Sukkar, S.G.; Cogorno, L.; Pisciotta, L.; Pasta, A.; Vena, A.; Gradaschi, R.; Dentone, C.; Guiddo, E.; Martino, E.; Beltramini, S.; et al. Clinical efficacy of eucaloric ketogenic nutrition in the COVID-19 cytokine storm: A retrospective analysis of mortality and intensive care unit admission. *Nutrition* **2021**, *89*, 111236, doi:10.1016/j.nut.2021.111236.
257. Sun, S.; Kong, Z.; Shi, Q.; Hu, M.; Zhang, H.; Di Zhang; Nie, J. Non-Energy-Restricted Low-Carbohydrate Diet Combined with Exercise Intervention Improved Cardiometabolic Health in Overweight Chinese Females. *Nutrients* **2019**, *11*, doi:10.3390/nu11123051.
258. Svart, M.; Gormsen, L.C.; Espersen, R.; Rittig, N.; Starup-Linde, J.; Møller, N.; Rejnmark, L. 3-Hydroxybutyrate administration elevates plasma parathyroid hormone in a pilot human randomized, controlled, cross over trial. *Bone* **2021**, *153*, 116166, doi:10.1016/j.bone.2021.116166.
259. Svedlund, A.; Hallböök, T.; Magnusson, P.; Dahlgren, J.; Swolin-Eide, D. Prospective study of growth and bone mass in Swedish children treated with the modified Atkins diet. *Eur. J. Paediatr. Neurol.* **2019**, *23*, 629–638, doi:10.1016/j.ejpn.2019.04.001.
260. Tay, J.; Thompson, C.H.; Luscombe-Marsh, N.D.; Noakes, M.; Buckley, J.D.; Wittert, G.A.; Brinkworth, G.D. Nutritional adequacy of very low- and high-carbohydrate, low saturated fat diets in adults with type 2 diabetes: A secondary analysis of a 2-year randomised controlled trial. *Diabetes Res. Clin. Pract.* **2020**, *170*, 108501, doi:10.1016/j.diabres.2020.108501.
261. Terink, R.; Witkamp, R.F.; Hopman, M.T.E.; Siebelink, E.; Savelkoul, H.F.J.; Mensink, M. A 2 Week Cross-over Intervention with a Low Carbohydrate, High Fat Diet Compared to a High Carbohydrate Diet Attenuates Exercise-Induced Cortisol Response, but Not the Reduction of Exercise Capacity, in Recreational Athletes. *Nutrients* **2021**, *13*, doi:10.3390/nu13010157.
262. Thompson, L.; Sullivan, D.K.; Varghese, K.; Abdelmoity, A.; Malik, M.; Abdelmoity, A.; Hall, A. Factors Associated With Growth in Patients Treated With the Classic Ketogenic Diet for Drug-Resistant Epilepsy. *Pediatr. Neurol.* **2023**, *146*, 110–115, doi:10.1016/j.pediatrneurol.2023.06.007.
263. Titcomb, T.J.; Liu, B.; Wahls, T.L.; Snetselaar, L.G.; Shadyab, A.H.; Tabung, F.K.; Saquib, N.; Arcan, C.; Tinker, L.F.; Wallace, R.B.; et al. Comparison of the Ketogenic Ratio of Macronutrients With the Low-Carbohydrate Diet Score and Their Association With Risk of Type 2 Diabetes in Postmenopausal Women: A Secondary Analysis of the Women's Health Initiative. *J. Acad. Nutr. Diet.* **2023**, *123*, 1152-1161.e4, doi:10.1016/j.jand.2022.12.004.
264. Turton, J.L.; Brinkworth, G.D.; Parker, H.M.; Lim, D.; Lee, K.; Rush, A.; Johnson, R.; Rooney, K.B. Effects of a low-carbohydrate diet in adults with type 1 diabetes management: A single arm non-randomised clinical trial. *PLoS One* **2023**, *18*, e0288440, doi:10.1371/journal.pone.0288440.
265. Tzenios, N.; Lewis, E.D.; Crowley, D.C.; Chahine, M.; Evans, M. Examining the Efficacy of a Very-Low-Carbohydrate Ketogenic Diet on Cardiovascular Health in Adults with Mildly Elevated Low-Density Lipoprotein Cholesterol in an Open-Label Pilot Study. *Metab. Syndr. Relat. Disord.* **2022**, *20*, 94–103, doi:10.1089/met.2021.0042.
266. Valinejad, A.; Khodaei, K. Does exercise during a ketogenic diet effectively alter appetite sensation, appetite-regulating hormones, and body composition? *Exp. Biol. Med. (Maywood)* **2022**, *247*, 1898–1906, doi:10.1177/15353702221113862.

267. van der Louw, E.; Olieman, J.; Poley, M.J.; Wesstein, T.; Vehmeijer, F.; Catsman-Berrevoets, C.; Neuteboom, R. Outpatient initiation of the ketogenic diet in children with pharmacoresistant epilepsy: An effectiveness, safety and economic perspective. *Eur. J. Paediatr. Neurol.* **2019**, *23*, 740–748, doi:10.1016/j.ejpn.2019.06.001.
268. Vandenberghe, C.; Castellano, C.-A.; Maltais, M.; Fortier, M.; St-Pierre, V.; Dionne, I.J.; Cunnane, S.C. A short-term intervention combining aerobic exercise with medium-chain triglycerides (MCT) is more ketogenic than either MCT or aerobic exercise alone: a comparison of normoglycemic and prediabetic older women. *Appl. Physiol. Nutr. Metab.* **2019**, *44*, 66–73, doi:10.1139/apnm-2018-0367.
269. Vargas-Molina, S.; Carbone, L.; Romance, R.; Petro, J.L.; Schoenfeld, B.J.; Kreider, R.B.; Bonilla, D.A.; Benítez-Porres, J. Effects of a low-carbohydrate ketogenic diet on health parameters in resistance-trained women. *Eur. J. Appl. Physiol.* **2021**, *121*, 2349–2359, doi:10.1007/s00421-021-04707-3.
270. Vargas-Molina, S.; García-Sillero, M.; Bonilla, D.A.; Petro, J.L.; García-Romero, J.; Benítez-Porres, J. The effect of the ketogenic diet on resistance training load management: a repeated-measures clinical trial in trained participants. *J. Int. Soc. Sports Nutr.* **2024**, *21*, 2306308, doi:10.1080/15502783.2024.2306308.
271. Vargas-Molina, S.; Petro, J.L.; Romance, R.; Kreider, R.B.; Schoenfeld, B.J.; Bonilla, D.A.; Benítez-Porres, J. Effects of a ketogenic diet on body composition and strength in trained women. *J. Int. Soc. Sports Nutr.* **2020**, *17*, 19, doi:10.1186/s12970-020-00348-7.
272. Vidić, V.; Ilić, V.; Toskić, L.; Janković, N.; Ugarković, D. Effects of calorie restricted low carbohydrate high fat ketogenic vs. non-ketogenic diet on strength, body-composition, hormonal and lipid profile in trained middle-aged men. *Clin. Nutr.* **2021**, *40*, 1495–1502, doi:10.1016/j.clnu.2021.02.028.
273. Vigh-Larsen, J.F.; Ørtenblad, N.; Nielsen, J.; Emil Andersen, O.; Overgaard, K.; Mohr, M. The Role of Muscle Glycogen Content and Localization in High-Intensity Exercise Performance: A Placebo-Controlled Trial. *Med. Sci. Sports Exerc.* **2022**, *54*, 2073–2086, doi:10.1249/MSS.0000000000003002.
274. Voils, C.I.; Pendergast, J.; Hale, S.L.; Gierisch, J.M.; Strawbridge, E.M.; Levine, E.; McVay, M.A.; Reed, S.D.; Yancy, W.S.; Shaw, R.J. A randomized feasibility pilot trial of a financial incentives intervention for dietary self-monitoring and weight loss in adults with obesity. *Transl. Behav. Med.* **2021**, *11*, 954–969, doi:10.1093/tbm/ibaa102.
275. Voss, M.; Wagner, M.; Mettenheim, N. von; Harter, P.N.; Wenger, K.J.; Franz, K.; Bojunga, J.; Vetter, M.; Gerlach, R.; Glatzel, M.; et al. ERGO2: A Prospective, Randomized Trial of Calorie-Restricted Ketogenic Diet and Fasting in Addition to Reirradiation for Malignant Glioma. *Int. J. Radiat. Oncol. Biol. Phys.* **2020**, *108*, 987–995, doi:10.1016/j.ijrobp.2020.06.021.
276. Voss, M.; Wenger, K.J.; Mettenheim, N. von; Bojunga, J.; Vetter, M.; Diehl, B.; Franz, K.; Gerlach, R.; Ronellenfitsch, M.W.; Harter, P.N.; et al. Short-term fasting in glioma patients: analysis of diet diaries and metabolic parameters of the ERGO2 trial. *Eur. J. Nutr.* **2022**, *61*, 477–487, doi:10.1007/s00394-021-02666-1.
277. Wachsmuth, N.B.; Aberer, F.; Haupt, S.; Schierbauer, J.R.; Zimmer, R.T.; Eckstein, M.L.; Zunner, B.; Schmidt, W.; Niedrist, T.; Sourij, H.; et al. The Impact of a High-Carbohydrate/Low Fat vs. Low-Carbohydrate Diet on Performance and Body Composition in Physically Active Adults: A Cross-Over Controlled Trial. *Nutrients* **2022**, *14*, doi:10.3390/nu14030423.
278. Wang, Y.; Jing, M.-X.; Jiang, L.; Jia, Y.-F.; Ying, E.; Cao, H.; Guo, X.-Y.; Sun, T. Does a ketogenic diet as an adjuvant therapy for drug treatment enhance chemotherapy sensitivity and reduce target lesions in patients with locally recurrent or metastatic Her-2-negative breast cancer? Study protocol for a randomized controlled trial. *Trials* **2020**, *21*, 487, doi:10.1186/s13063-020-04429-5.
279. White, H.; Venkatesh, B.; Jones, M.; Kruger, P.S.; Walsham, J.; Fuentes, H. Inducing ketogenesis via an enteral formulation in patients with acute brain injury:a phase II study. *Neurol. Res.* **2020**, *42*, 275–285, doi:10.1080/01616412.2019.1709743.
280. Wilson, J.M.; Lowery, R.P.; Roberts, M.D.; Sharp, M.H.; Joy, J.M.; Shields, K.A.; Partl, J.M.; Volek, J.S.; D'Agostino, D.P. Effects of Ketogenic Dieting on Body Composition, Strength, Power, and Hormonal Profiles in Resistance Training Men. *J. Strength Cond. Res.* **2020**, *34*, 3463–3474, doi:10.1519/JSC.0000000000001935.
281. Woelber, J.P.; Tennert, C.; Ernst, S.F.; Vach, K.; Ratka-Krüger, P.; Bertz, H.; Urbain, P. Effects of a Non-Energy-Restricted Ketogenic Diet on Clinical Oral Parameters. An Exploratory Pilot Trial. *Nutrients* **2021**, *13*, doi:10.3390/nu13124229.
282. Wong, J.M.W.; Ludwig, D.S.; Allison, D.B.; Baidwan, N.; Bielak, L.; Chiu, C.-Y.; Dickinson, S.L.; Golzarri-Arroyo, L.; Heymsfield, S.B.; Holmes, L.; et al. Design and conduct of a randomized controlled feeding trial

in a residential setting with mitigation for COVID-19. *Contemp. Clin. Trials* **2024**, *140*, 107490, doi:10.1016/j.cct.2024.107490.
283. Wroble, K.A.; Trott, M.N.; Schweitzer, G.G.; Rahman, R.S.; Kelly, P.V.; Weiss, E.P. Low-carbohydrate, ketogenic diet impairs anaerobic exercise performance in exercise-trained women and men: a randomized-sequence crossover trial. *J. Sports Med. Phys. Fitness* **2019**, *59*, 600–607, doi:10.23736/S0022-4707.18.08318-4.
284. Wu, W.; Zhou, Q.; Yuan, P.; Qiao, D.; Deng, S.; Cheng, H.; Ren, Y. A Novel Multiphase Modified Ketogenic Diet: An Effective and Safe Tool for Weight Loss in Chinese Obese Patients. *Diabetes Metab. Syndr. Obes.* **2022**, *15*, 2521–2534, doi:10.2147/DMSO.S365192.
285. Xu, Q.; Zhang, Y.; Zhang, X.; Liu, L.; Zhou, B.; Mo, R.; Li, Y.; Li, H.; Li, F.; Tao, Y.; et al. Medium-chain triglycerides improved cognition and lipid metabolomics in mild to moderate Alzheimer's disease patients with APOE4-/-: A double-blind, randomized, placebo-controlled crossover trial. *Clin. Nutr.* **2020**, *39*, 2092–2105, doi:10.1016/j.clnu.2019.10.017.
286. Yancy, W.S.; Crowley, M.J.; Dar, M.S.; Coffman, C.J.; Jeffreys, A.S.; Maciejewski, M.L.; Voils, C.I.; Bradley, A.B.; Edelman, D. Comparison of Group Medical Visits Combined With Intensive Weight Management vs Group Medical Visits Alone for Glycemia in Patients With Type 2 Diabetes: A Noninferiority Randomized Clinical Trial. *JAMA Intern. Med.* **2020**, *180*, 70–79, doi:10.1001/jamainternmed.2019.4802.
287. Yang, M.; Bai, W.; Jiang, B.; Wang, Z.; Wang, X.; Sun, Y.; Liu, F.; Cui, G.; Song, X.; Li, J.; et al. Effects of a ketogenic diet in women with PCOS with different uric acid concentrations: a prospective cohort study. *Reprod. Biomed. Online* **2022**, *45*, 391–400, doi:10.1016/j.rbmo.2022.03.023.
288. Yomogida, Y.; Matsuo, J.; Ishida, I.; Ota, M.; Nakamura, K.; Ashida, K.; Kunugi, H. An fMRI Investigation into the Effects of Ketogenic Medium-Chain Triglycerides on Cognitive Function in Elderly Adults: A Pilot Study. *Nutrients* **2021**, *13*, doi:10.3390/nu13072134.
289. Yu, L.F.; Zhang, Y.Q.; Duan, J.; Ni, Y.; Gong, X.Y.; Lu, Z.Y.; Liao, J.X.; Lu, X.P.; Shi, Z.N.; Lei, M.F.; et al. Clinical characteristics and ketogenic diet therapy of glucose transporter type 1 deficiency syndrome in children: a multicenter clinical study. *Zhonghua Er Ke Za Zhi* **2020**, *58*, 881–886, doi:10.3760/cma.j.cn112140-20200822-00819.
290. Zainordin, N.A.; Eddy Warman, N.A.; Mohamad, A.F.; Abu Yazid, F.A.; Ismail, N.H.; Chen, X.W.; Koshy, M.; Abdul Rahman, T.H.; Mohd Ismail, N.; Abdul Ghani, R. Safety and efficacy of very low carbohydrate diet in patients with diabetic kidney disease-A randomized controlled trial. *PLoS One* **2021**, *16*, e0258507, doi:10.1371/journal.pone.0258507.
